# Supplementary material for: Promoting engagement with social fact-checks online: Investigating the roles of social connection and shared partisanship
Source: PLoS One. 2025 Mar 31;20(3):e0319336. doi: 10.1371/journal.pone.0319336 (PMC11957346; doi:10.1371/journal.pone.0319336)
Supplement: S1 Text — Supporting information for “Promoting engagement with social fact-checks online: Investigating the roles of social connection and shared partisanship”. (PDF) [file pone.0319336.s001.pdf]

Supporting Information for

Promoting engagement with social fact-checks online: Investigating the roles of social connection and shared partisanship

Martel, Mosleh *et al.*
*Corresponding author. Email: cmartel@mit.edu

SI Table of Contents.

1. **Main Text Survey Experiment 1**
   a. **Pre-registration.**
   b. **Descriptive Statistics.**
   c. **Main Text Analyses.**
   d. **Main Text Analyses – Binary Candidate Choice Partisanship.**
   e. **Pre-registered Analyses.**
   f. **Bayesian Analyses.**
   g. **False Discovery Rate Analyses.**
2. **Main Text Survey Experiment 2**
   a. **Pre-registration.**
   b. **Descriptive Statistics.**
   c. **Main Analyses.**
   d. **Bayesian Analyses.**
   e. **Ordinal Analyses.**
3. **Supplemental Survey Experiment S1**
   a. **Pre-registration.**
   b. **Descriptive Statistics.**
   c. **Main Analyses.**
   d. **Pre-registered Secondary Analyses.**
   e. **Combined Survey Experiment 1 & Supplemental Survey Experiment S1: Exploratory Analysis.**
   f. **Bayesian Analyses.**
4. **Twitter (X) Field Experiment**
   a. **Materials.**
   b. **Descriptive Statistics.**
   c. **Pre-registered Analyses.**
   d. **Full Main Analyses – Messaged Users Only.**
   e. **Bayesian Analyses.**
   f. **Reply Tweet Rating.**
   g. **Supplementary Results Text.**
   h. **Follow-back Analyses.**
5. **Partisan Extremity Analyses: Field Experiment & Main Survey Experiment 1**
   a. **Relationship between Partisan Extremity and Correction Delivery Failure (i.e., Blocking) in Field Experiment.**
   b. **User Partisan Extremity in Field Experiment.**
   c. **Participant Partisan Extremity in Main Survey Experiment 1**
6. **References**

Note: Unless otherwise stated, the following applies for all supplementary tables:
**p*<.05, ***p*<.01, ****p*<.001

1. Main Text Survey Experiment 1

a. Pre-registration.

In our pre-registration (https://aspredicted.org/ZHT_SQW), we specified our main analysis as a regression model predicting key dependent variables (engagement; various mechanism questions) by social condition (-0.5 = baseline, 0.5 = social), political concordance (i.e., shared partisanship; -0.5=counter-partisan, 0.5=co-partisan), and their interaction. As our main reported analyses, we deviate from this pre-registration plan to also include a *z*-scored continuous measure of partisanship as a predictor, allowing for all interactions. However, we also report full analyses and results here for these originally pre-registered analyses.

b. Descriptive Statistics.

Supplementary Table 1. Reply engagement descriptive statistics by condition. Total N=808.

Baseline, Counter-partisan	Baseline, Co-partisan	Social, Counter-partisan	Social, Co-partisan
Engaged: 102<br>Ignored: 100<br>M_{engage}: 50.50%	Engaged: 105<br>Ignored: 99<br>M_{engage}: 51.47%	Engaged: 107<br>Ignored: 89<br>M_{engage}: 54.59%	Engaged: 133<br>Ignored: 73<br>M_{engage}: 64.56%

c. Main Text Analyses.

Supplementary Table 2. Reply engagement (binary) predicted by social condition, participant partisanship, shared partisanship, and all interactions.

	b	*Std*	*t*	*p*
(Intercept)	0.553	0.017	31.828	<0.001***
Social condition	0.083	0.035	2.373	0.018*
Participant partisanship	-0.048	0.017	-2.739	0.006**
Shared partisanship	0.028	0.017	1.608	0.108
Social condition × Participant partisanship	0.001	0.035	0.039	0.969
Social condition × Shared partisanship	0.043	0.035	1.242	0.214
Participant partisanship × Shared partisanship	-0.012	0.017	-0.699	0.485
Social condition × Participant partisanship × Shared partisanship	0.024	0.035	0.687	0.492

Supplementary Table 3. Rudeness of non-responding predicted by social condition, participant partisanship, shared partisanship, and all interactions.

	b	*Std*	*t*	*p*
(Intercept)	3.296	0.072	45.857	<0.001***
Social condition	0.174	0.144	1.208	0.227
Participant partisanship	-0.152	0.072	-2.101	0.036*
Shared partisanship	0.019	0.072	0.261	0.794
Social condition × Participant partisanship	-0.064	0.144	-0.445	0.656

Social condition ⨉ Shared partisanship	0.067	0.144	0.467	0.640
Participant partisanship ⨉ Shared partisanship	-0.062	0.072	-0.859	0.391
Social condition ⨉ Participant partisanship ⨉ Shared partisanship	-0.015	0.144	-0.107	0.915

Supplementary Table 4. Obligation to respond predicted by social condition, participant partisanship, shared partisanship, and all interactions.

	b	*Std*	*t*	*p*
(Intercept)	3.751	0.074	50.882	<0.001***
Social condition	0.3	0.147	2.031	0.043*
Participant partisanship	-0.142	0.074	-1.921	0.055
Shared partisanship	0.005	0.074	0.074	0.941
Social condition ⨉ Participant partisanship	-0.015	0.148	-0.104	0.917
Social condition ⨉ Shared partisanship	0.07	0.148	0.476	0.634
Participant partisanship ⨉ Shared partisanship	-0.056	0.074	-0.759	0.448
Social condition ⨉ Participant partisanship ⨉ Shared partisanship	-0.042	0.148	-0.282	0.778

Supplementary Table 5. Appropriateness of correcting others predicted by social condition, participant partisanship, shared partisanship, and all interactions.

	b	***Std***	***t***	***p***
(Intercept)	5.168	0.041	125.694	<0.001***
Social condition	0.074	0.082	0.899	0.369
Participant partisanship	-0.169	0.041	-4.096	<0.001***
Shared partisanship	0.022	0.041	0.544	0.587
Social condition ⨉ Participant partisanship	0.098	0.082	1.182	0.237
Social condition ⨉ Shared partisanship	-0.02	0.082	-0.243	0.808
Participant partisanship ⨉ Shared partisanship	-0.002	0.041	-0.054	0.957
Social condition ⨉ Participant partisanship ⨉ Shared partisanship	0.174	0.083	2.108	0.035*

Supplementary Table 6. Comfort with corrector (reverse-coded) predicted by social condition, participant partisanship, shared partisanship, and all interactions.

	b	***Std***	***t***	***p***
(Intercept)	3.798	0.06	62.809	<0.001***
Social condition	-0.031	0.121	-0.254	0.799

Participant partisanship	-0.095	0.061	-1.571	0.117
Shared partisanship	-0.113	0.061	-1.862	0.063
Social condition × Participant partisanship	-0.051	0.121	-0.419	0.675
Social condition × Shared partisanship	-0.124	0.121	-1.024	0.306
Participant partisanship × Shared partisanship	-0.053	0.061	-0.869	0.385
Social condition × Participant partisanship × Shared partisanship	0.111	0.121	0.91	0.363

Supplementary Table 7. Trustworthiness of corrector predicted by social condition, participant partisanship, shared partisanship, and all interactions.

	b	***Std***	***t***	***p***
(Intercept)	4.705	0.048	98.269	<0.001***
Social condition	0.092	0.096	0.962	0.336
Participant partisanship	-0.13	0.048	-2.7	0.007**
Shared partisanship	0.117	0.048	2.444	0.015*
Social condition × Participant partisanship	0.089	0.096	0.928	0.354
Social condition × Shared partisanship	0.043	0.096	0.446	0.656
Participant partisanship × Shared partisanship	-0.062	0.048	-1.3	0.194
Social condition × Participant partisanship × Shared partisanship	0.048	0.096	0.494	0.621

Supplementary Table 8. How smart is corrector predicted by social condition, participant partisanship, shared partisanship, and all interactions.

	b	***Std***	***t***	***p***
(Intercept)	4.988	0.045	112.044	<0.001***
Social condition	0.132	0.089	1.486	0.138
Participant partisanship	-0.125	0.045	-2.8	0.005**
Shared partisanship	0.114	0.045	2.568	0.010**
Social condition × Participant partisanship	0.059	0.089	0.663	0.508
Social condition × Shared partisanship	0.014	0.089	0.159	0.874
Participant partisanship × Shared partisanship	-0.066	0.045	-1.467	0.143
Social condition × Participant partisanship × Shared partisanship	0.082	0.089	0.922	0.357

Supplementary Table 9. Positive/negative feelings towards corrector predicted by social condition, participant partisanship, shared partisanship, and all interactions.

	b	***Std***	***t***	***p***
(Intercept)	4.69	0.048	98.01	<0.001***
Social condition	0.141	0.096	1.473	0.141
Participant partisanship	-0.12	0.048	-2.505	0.012*
Shared partisanship	0.154	0.048	3.216	0.001***
Social condition ⨉ Participant partisanship	0.109	0.096	1.138	0.255
Social condition ⨉ Shared partisanship	0.115	0.096	1.196	0.232
Participant partisanship ⨉ Shared partisanship	-0.096	0.048	-1.987	0.047*
Social condition ⨉ Participant partisanship ⨉ Shared partisanship	0.023	0.096	0.234	0.815

Supplementary Table 10. Likelihood corrector is bot predicted by social condition, participant partisanship, shared partisanship, and all interactions.

	b	***Std***	***t***	***p***
(Intercept)	4.427	0.06	73.483	<0.001***
Social condition	-0.002	0.12	-0.014	0.989
Participant partisanship	-0.192	0.06	-3.182	0.002**
Shared partisanship	-0.028	0.06	-0.465	0.642
Social condition ⨉ Participant partisanship	-0.094	0.121	-0.775	0.439
Social condition ⨉ Shared partisanship	-0.072	0.121	-0.595	0.552
Participant partisanship ⨉ Shared partisanship	0.038	0.061	0.628	0.530
Social condition ⨉ Participant partisanship ⨉ Shared partisanship	-0.039	0.121	-0.326	0.744

Supplementary Table 11. Obligated self-correction (exploratory) predicted by social condition, participant partisanship, shared partisanship, and all interactions.

	b	***Std***	***t***	***p***
(Intercept)	4.688	0.062	75.578	<0.001***
Social condition	0.066	0.124	0.528	0.597
Participant partisanship	-0.239	0.062	-3.834	<0.001***
Shared partisanship	0.043	0.062	0.686	0.493
Social condition ⨉ Participant partisanship	0.082	0.124	0.658	0.511
Social condition ⨉ Shared partisanship	0.009	0.124	0.073	0.942

Participant partisanship × Shared partisanship	-0.026	0.062	-0.421	0.674
Social condition × Participant partisanship × Shared partisanship	0.014	0.125	0.114	0.910

d. Main Text Analyses – Binary Candidate Choice Partisanship.

In addition to primarily assessing partisanship via a 1-7 Likert-scale, we also included a binary choice Presidential candidate measure of partisan identity. Here, we repeat our main analyses except replacing our continuous measure of partisanship with this binary choice measure of candidate preference. Crucially, we again observe a significant main effect of prior social connection increasing likelihood of responding to a correction message; we again do not observe an effect of shared partisanship on reply likelihood, nor do we observe an interaction between prior social connection and shared partisanship on reply likelihood.

Supplementary Table 12. Reply engagement (binary) predicted by social condition, participant partisanship, shared partisanship, and all interactions.

	b	***Std***	***t***	***p***
(Intercept)	0.553	0.017	32.265	<0.001***
Social condition	0.089	0.034	2.604	0.009**
Participant partisanship	0.091	0.017	5.330	<0.001***
Shared partisanship	0.015	0.017	0.882	0.378
Social condition × Participant partisanship	-0.018	0.034	-0.524	0.600
Social condition × Shared partisanship	0.028	0.034	0.812	0.417
Participant partisanship × Shared partisanship	-0.034	0.017	-1.982	0.048*
Social condition × Participant partisanship × Shared partisanship	-0.006	0.034	-0.168	0.866

Supplementary Table 13. Rudeness of non-responding predicted by social condition, participant partisanship, shared partisanship, and all interactions.

	b	***Std***	***t***	***p***
(Intercept)	3.297	0.067	49.133	<0.001***
Social condition	0.200	0.134	1.494	0.136
Participant partisanship	0.740	0.067	11.025	<0.001***
Shared partisanship	-0.011	0.067	-0.169	0.866
Social condition × Participant partisanship	-0.149	0.134	-1.111	0.267
Social condition × Shared partisanship	-0.007	0.134	-0.054	0.957
Participant partisanship × Shared partisanship	-0.043	0.067	-0.641	0.521
Social condition × Participant partisanship × Shared partisanship	-0.040	0.134	-0.297	0.766

Supplementary Table 14. Obligation to respond predicted by social condition, participant partisanship, shared partisanship, and all interactions.

	b	***Std***	***t***	***p***
(Intercept)	3.753	0.068	54.86	<0.001***
Social condition	0.329	0.137	2.405	0.016*
Participant partisanship	0.784	0.068	11.445	<0.001***
Shared partisanship	-0.001	0.068	-0.014	0.988
Social condition × Participant partisanship	0.029	0.137	0.211	0.833
Social condition × Shared partisanship	0.018	0.137	0.130	0.897
Participant partisanship × Shared partisanship	-0.072	0.068	-1.054	0.292
Social condition × Participant partisanship × Shared partisanship	-0.140	0.137	-1.024	0.306

Supplementary Table 15. Appropriateness of correcting others predicted by social condition, participant partisanship, shared partisanship, and all interactions.

	b	***Std***	***t***	***p***
(Intercept)	5.165	0.042	124.195	<0.001***
Social condition	0.088	0.083	1.061	0.289
Participant partisanship	0.071	0.042	1.701	0.089
Shared partisanship	0.037	0.042	0.893	0.372
Social condition × Participant partisanship	0.063	0.083	0.753	0.451
Social condition × Shared partisanship	0.016	0.083	0.189	0.850
Participant partisanship × Shared partisanship	0.004	0.042	0.104	0.917
Social condition × Participant partisanship × Shared partisanship	0.029	0.083	0.347	0.729

Supplementary Table 16. Comfort with corrector (reverse-coded) predicted by social condition, participant partisanship, shared partisanship, and all interactions.

	b	***Std***	***t***	***p***
(Intercept)	3.798	0.061	62.71	<0.001***
Social condition	-0.023	0.121	-0.186	0.853
Participant partisanship	-0.043	0.061	-0.706	0.480
Shared partisanship	-0.112	0.061	-1.848	0.065
Social condition × Participant partisanship	0.015	0.121	0.124	0.902

Social condition × Shared partisanship	-0.076	0.121	-0.625	0.532
Participant partisanship × Shared partisanship	0.005	0.061	0.088	0.930
Social condition × Participant partisanship × Shared partisanship	0.128	0.121	1.058	0.290

Supplementary Table 17. Trustworthiness of corrector predicted by social condition, participant partisanship, shared partisanship, and all interactions.

	b	***Std***	***t***	***p***
(Intercept)	4.703	0.047	100.515	<0.001***
Social condition	0.109	0.094	1.170	0.242
Participant partisanship	0.268	0.047	5.720	<0.001***
Shared partisanship	0.156	0.047	3.340	0.001***
Social condition × Participant partisanship	0.063	0.094	0.678	0.498
Social condition × Shared partisanship	0.149	0.094	1.596	0.111
Participant partisanship × Shared partisanship	-0.123	0.047	-2.632	0.009**
Social condition × Participant partisanship × Shared partisanship	0.008	0.094	0.083	0.934

Supplementary Table 18. How smart is corrector predicted by social condition, participant partisanship, shared partisanship, and all interactions.

	b	***Std***	***t***	***p***
(Intercept)	4.987	0.043	114.863	<0.001***
Social condition	0.151	0.087	1.735	0.083
Participant partisanship	0.280	0.043	6.447	<0.001***
Shared partisanship	0.115	0.043	2.647	0.008**
Social condition × Participant partisanship	0.093	0.087	1.068	0.286
Social condition × Shared partisanship	0.063	0.087	0.729	0.466
Participant partisanship × Shared partisanship	-0.126	0.043	-2.899	0.004**
Social condition × Participant partisanship × Shared partisanship	0.062	0.087	0.719	0.473

Supplementary Table 19. Positive/negative feelings towards corrector predicted by social condition, participant partisanship, shared partisanship, and all interactions.

	b	***Std***	***t***	***p***
(Intercept)	4.689	0.046	101.933	<0.001***

Social condition	0.162	0.092	1.761	0.079
Participant partisanship	0.353	0.046	7.671	<0.001***
Shared partisanship	0.157	0.046	3.414	0.001***
Social condition × Participant partisanship	0.143	0.092	1.549	0.122
Social condition × Shared partisanship	0.161	0.092	1.752	0.080
Participant partisanship × Shared partisanship	-0.169	0.046	-3.676	<0.001***
Social condition × Participant partisanship × Shared partisanship	-0.076	0.092	-0.829	0.408

Supplementary Table 20. Likelihood corrector is bot predicted by social condition, participant partisanship, shared partisanship, and all interactions.

	b	***Std***	***t***	***p***
(Intercept)	4.430	0.058	76.405	<0.001***
Social condition	0.020	0.116	0.171	0.864
Participant partisanship	0.492	0.058	8.476	<0.001***
Shared partisanship	-0.025	0.058	-0.425	0.671
Social condition × Participant partisanship	0.131	0.116	1.132	0.258
Social condition × Shared partisanship	-0.027	0.116	-0.233	0.816
Participant partisanship × Shared partisanship	0.077	0.058	1.321	0.187
Social condition × Participant partisanship × Shared partisanship	0.031	0.116	0.268	0.789

Supplementary Table 21. Obligated self-correction (exploratory) predicted by social condition, participant partisanship, shared partisanship, and all interactions.

	b	***Std***	***t***	***p***
(Intercept)	4.686	0.062	75.216	<0.001***
Social condition	0.090	0.125	0.722	0.471
Participant partisanship	0.144	0.062	2.317	0.021*
Shared partisanship	0.033	0.062	0.530	0.596
Social condition × Participant partisanship	-0.022	0.125	-0.180	0.857
Social condition × Shared partisanship	0.059	0.125	0.476	0.635
Participant partisanship × Shared partisanship	-0.066	0.062	-1.057	0.291
Social condition × Participant partisanship × Shared partisanship	-0.113	0.125	-0.906	0.365

e. Pre-registered Analyses.

Survey experiment analyses conducted exactly as pre-registered.

Supplementary Table 22. Reply engagement (binary) predicted by social condition, shared partisanship, and their interaction.

	b	***Std***	***t***	***p***
(Intercept)	0.553	0.017	31.752	<0.001***
Social condition	0.087	0.035	2.486	0.013*
Shared partisanship	0.027	0.017	1.571	0.117
Social condition × Shared partisanship	0.045	0.035	1.291	0.197

Supplementary Table 23. Rudeness of non-responding predicted by social condition, shared partisanship, and their interaction

	b	***Std***	***t***	***p***
(Intercept)	3.298	0.072	45.872	<0.001***
Social condition	0.187	0.144	1.298	0.195
Shared partisanship	0.019	0.072	0.267	0.790
Social condition × Shared partisanship	0.075	0.144	0.521	0.603

Supplementary Table 24. Obligation to respond predicted by social condition, shared partisanship, and their interaction.

	b	***Std***	***t***	***p***
(Intercept)	3.752	0.074	50.919	<0.001***
Social condition	0.312	0.147	2.116	0.035*
Shared partisanship	0.006	0.074	0.082	0.935
Social condition × Shared partisanship	0.077	0.147	0.523	0.601

Supplementary Table 25. Appropriateness of correcting others predicted by social condition, shared partisanship, and their interaction.

	b	***Std***	***t***	***p***
(Intercept)	5.165	0.042	124.205	<0.001***
Social condition	0.088	0.083	1.057	0.291
Shared partisanship	0.018	0.042	0.436	0.663
Social condition × Shared partisanship	-0.017	0.083	-0.202	0.840

Supplementary Table 26. Comfort with corrector (reverse-coded) predicted by social condition, shared partisanship, and their interaction.

	b	***Std***	***t***	***p***
(Intercept)	3.799	0.06	62.855	<0.001***

Social condition	-0.022	0.121	-0.182	0.856
Shared partisanship	-0.115	0.06	-1.899	0.058
Social condition × Shared partisanship	-0.118	0.121	-0.978	0.329

Supplementary Table 27. Trustworthiness of corrector predicted by social condition, shared partisanship, and their interaction.

	b	***Std***	***t***	***p***
(Intercept)	4.703	0.048	97.893	<0.001***
Social condition	0.104	0.096	1.079	0.281
Shared partisanship	0.115	0.048	2.403	0.017*
Social condition × Shared partisanship	0.05	0.096	0.521	0.602

Supplementary Table 28. How smart is corrector predicted by social condition, shared partisanship, and their interaction.

	b	***Std***	***t***	***p***
(Intercept)	4.987	0.045	111.539	<0.001***
Social condition	0.144	0.089	1.607	0.108
Shared partisanship	0.112	0.045	2.509	0.012*
Social condition × Shared partisanship	0.022	0.089	0.241	0.810

Supplementary Table 29. Positive/negative feelings towards corrector predicted by social condition, shared partisanship, and their interaction.

	b	***Std***	***t***	***p***
(Intercept)	4.688	0.048	97.531	<0.001***
Social condition	0.152	0.096	1.584	0.114
Shared partisanship	0.153	0.048	3.178	0.002**
Social condition × Shared partisanship	0.124	0.096	1.293	0.196

Supplementary Table 30. Likelihood corrector is bot predicted by social condition, shared partisanship, and their interaction.

	b	***Std***	***t***	***p***
(Intercept)	4.429	0.06	73.266	<0.001***
Social condition	0.014	0.121	0.113	0.910
Shared partisanship	-0.027	0.06	-0.446	0.656
Social condition × Shared partisanship	-0.072	0.121	-0.598	0.550

Supplementary Table 31. Obligated self-correction (exploratory) predicted by social condition, shared partisanship, and their interaction.

	b	***Std***	***t***	***p***
(Intercept)	4.686	0.062	75.062	<0.001***

Social condition	0.085	0.125	0.682	0.495
Shared partisanship	0.041	0.062	0.663	0.507
Social condition X Shared partisanship	0.015	0.125	0.122	0.903

f. Bayesian Analyses

Supplementary Table 32. Main survey experiment 1 Bayesian regression analysis of effect of prior social connection, participant partisanship, and shared partisanship between participant and corrector on engagement using weakly-informative normal prior over intercept and effect of predictors and weakly-informative prior over residual standard deviation. l-95% CI shows the 0.025 percentile of posterior distribution and u-95% CI shows the 0.975 percentile of posterior distribution.

	Estimate	Estimate err.	l-95%	u-95%	$\hat{R}$	Bulk ESS	Tail ESS
(Intercept)	0.55	0.02	0.52	0.59	1	14003	5445
Social condition	0.08	0.03	0.01	0.15	1	13716	6365
Participant partisanship	-0.05	0.02	-0.08	-0.01	1	14254	5609
Shared partisanship	0.03	0.02	-0.01	0.06	1	15056	6359
Social condition X Participant partisanship	0	0.04	-0.07	0.07	1	15159	5616
Social condition X Shared partisanship	0.04	0.04	-0.03	0.11	1	13272	6445
Participant partisanship X Shared partisanship	-0.01	0.02	-0.05	0.02	1	13271	6471
Social condition X Participant partisanship X Shared partisanship	0.02	0.04	-0.04	0.09	1	14416	6033

g. False Discovery Rate Analyses.

We found preliminary evidence that participants in the social condition felt more obligated to reply to the corrective message than participants in the baseline condition, $b = 0.300$, $SE = 0.147$, $t(807) = 2.031$, $p = .043$. We next correct for multiple comparisons by calculating q-values, which indicate the probability of making at least one false discovery across all comparisons. We first computed a calculated q-value for obligated responding as 1-(1-p)^n, where n is the number of comparisons for main effects of prior social connection on mechanism (i.e., n=8; 8 mechanisms, not including obligated self-correction (exploratory), and $p = .0426$). This calculated q-value is 0.294 (see OSF analysis file https://osf.io/nvk4u/?view_only=2ba8efe34cd647e18f69857bd1f85bbd). Second, we computed a simulated q-value, which accounts for the fact that the assumption of independence between comparisons may be overly conservative. We simulate the expected probability of making at least one false discovery over 10,000 iterations, following the procedure of (1). Each iteration includes randomly sampling observations with replacement, randomly assigning observations to conditions, and conducting all relevant comparison tests. Then simulated q-value is computed as the proportion of iterations in which the minimum simulated p-value is less than the given p-value. Our simulated q-value was 0.225 (see OSF analysis file https://osf.io/nvk4u/?view_only=2ba8efe34cd647e18f69857bd1f85bbd for full simulation).

The effect of social condition on feeling obligated to reply was no longer significant after adjusting for multiple comparisons (calculated q-value = 0.294; simulated q-value = 0.225). We did not observe any difference between the social and baseline condition for any of our other potential mechanism variables, ps > .138.

Similar false discovery rate analyses were performed to examine the effect of shared partisanship on positive (versus negative) feelings towards the corrector; perceived corrector trustworthiness; and perceived corrector intelligence. Interestingly, we do observe an effect of shared partisanship on positive (versus negative) feelings towards the corrector ($p = .001$, calculated q-value = .011, simulated q-value = .009) even though we do not find an effect of shared partisanship on probability of responding. This further demonstrates the apparent disassociation between promoting engagement and fostering positive feelings towards the corrector – as minimal social connection appears to increase engagement but not positive sentiment, whereas shared partisanship may increase positive sentiment without increasing engagement. We also find some suggestive evidence of effects of shared partisanship on

perceived corrector trustworthiness (*p*= .015, calculated q-value = .112, simulated q-value = .091) and intelligence (*p* = .010, calculated q-value = .080, simulated q-value = .065), though these latter effects are no longer significant after adjusting for multiple comparisons. Ultimately, these results suggest that social connection is perhaps promoting engagement via a mechanism more closely related to establishing a norm or obligation to respond, rather than enhancing positive feelings or affect towards the corrector; whereas shared partisanship may promote the latter, without necessarily increasing engagement.

Given that the effect of prior social connection on obligation to respond in our first survey experiment was no longer significant after adjusting for multiple comparisons, we next conducted a follow-up survey experiment to further examine obligation to respond as a mechanism behind the relationship between minimal social relationships and engaging with correction messages.

2. Main Text Survey Experiment 2

a. Pre-registration.

We pre-registered this study here (https://aspredicted.org/G8Q_413). We made no deviations from our pre-registration.

b. Descriptive Statistics.

Supplementary Table 33. Personal obligation to respond descriptive statistics by condition (N=812).

Baseline, Non-correction	Baseline, Correction	Social, Non-correction	Social, Correction
N = 229<br>$M_{obligated}$ = 3.45<br>$SD_{obligated}$ = 2.22	N = 191<br>$M_{obligated}$ = 3.58<br>$SD_{obligated}$ = 2.08	N = 194<br>$M_{obligated}$ = 4.12<br>$SD_{obligated}$ = 2.04	N = 198<br>$M_{obligated}$ = 4.09<br>$SD_{obligated}$ = 2.08

Supplementary Table 34. Perceptions of reciprocal responding norm descriptive statistics by condition (N=794).

Baseline, Non-correction	Baseline, Correction	Social, Non-correction	Social, Correction
N = 185<br>M_{norm} = 4.44<br>SD_{norm} = 1.94	N = 197<br>M_{norm} = 4.81<br>SD_{norm}= 1.65	N = 204<br>M_{norm} = 4.72<br>SD_{norm} = 1.67	N = 208<br>M_{norm} = 5.03<br>SD_{norm} = 1.56

c. Main Analyses.

Supplementary Table 35. Personal obligation to respond predicted by social condition, correction condition, and interaction.

	b	***Std***	***t***	***p***
(Intercept)	3.809	0.074	51.285	<0.001***
Social condition	0.592	0.149	3.985	<0.001***
Correction condition	0.049	0.149	0.332	0.740
Social condition × Correction condition	-0.154	0.297	-0.518	0.605

Supplementary Table 36. Perceived norm of responding predicted by social condition, correction condition, and interaction.

	b	***Std***	***t***	***p***
(Intercept)	4.75	0.061	78.415	<0.001***
Social condition	0.245	0.121	2.019	0.044*
Correction condition	0.341	0.121	2.815	0.005**

Social condition × Correction condition	-0.056	0.242	-0.23	0.818

Supplementary Table 37. Personal obligation to respond predicted by social condition, correction condition, partisanship, and all interactions.

	b	***Std***	***t***	***p***
(Intercept)	4.453	0.141	31.505	<0.001***
Social condition	0.556	0.283	1.965	0.050*
Correction condition	0.322	0.283	1.14	0.254
Participant partisanship	-0.215	0.04	-5.326	<0.001***
Social condition × Correction condition	-0.23	0.565	-0.406	0.684
Social condition × Participant partisanship	-0.005	0.081	-0.065	0.948
Correction condition × Participant partisanship	-0.079	0.081	-0.979	0.328
Social condition × Correction condition × Participant partisanship	0.009	0.162	0.058	0.953

Supplementary Table 38. Perceived norm of responding predicted by social condition, correction condition, partisanship, and all interactions.

	b	***Std***	***t***	***p***
(Intercept)	5.07	0.115	44.232	<0.001***
Social condition	0.194	0.229	0.847	0.397
Correction condition	0.117	0.229	0.511	0.609
Participant partisanship	-0.107	0.032	-3.344	0.001***
Social condition × Correction condition	-0.629	0.459	-1.372	0.171
Social condition × Participant partisanship	0.018	0.064	0.285	0.775
Correction condition × Participant partisanship	0.081	0.064	1.253	0.211
Social condition × Correction condition × Participant partisanship	0.182	0.129	1.419	0.156

d. Bayesian Analyses.

Supplementary Table 39. Main survey experiment 2 Bayesian regression analysis of effect of prior social connection, type of response (non-correction, correction), and their interaction on personal obligation to respond using weakly-informative normal prior over intercept and effect of predictors and weakly-informative prior over residual standard deviation. l-95% CI shows the 0.025 percentile of posterior distribution and u-95% CI shows the 0.975 percentile of posterior distribution.

	**Estimate**	**Estimate err.**	**l-95%**	**u-95%**	$\hat{R}$	**Bulk ESS**	**Tail ESS**
(Intercept)	3.54	0.07	3.39	3.68	1	9637	5865
Social condition	0.58	0.15	0.29	0.87	1	10738	5580

Correction response	0.05	0.15	-0.24	0.34	1	9038	5974
Social condition X Correction response	-0.14	0.29	-0.7	0.42	1	10521	6078

Supplementary Table 40. Main survey experiment 2 Bayesian regression analysis of effect of prior social connection, type of response (non-correction, correction), and their interaction on perceived norm of responding using weakly-informative normal prior over intercept and effect of predictors and weakly-informative prior over residual standard deviation. l-95% CI shows the 0.025 percentile of posterior distribution and u-95% CI shows the 0.975 percentile of posterior distribution.

	**Estimate**	**Estimate err.**	**l-95%**	**u-95%**	$\hat{R}$	**Bulk ESS**	**Tail ESS**
(Intercept)	4.51	0.06	4.39	4.63	1	9743	5951
Social condition	0.24	0.12	0.01	0.48	1	9676	6327
Correction response	0.34	0.12	0.1	0.57	1	9748	6203
Social condition X Correction response	-0.05	0.24	-0.52	0.4	1	9731	5967

e. Ordinal Analyses.

Supplementary Table 35b. Personal obligation to respond predicted by social condition, correction condition, and interaction – ordinal logistic regression.

	b	***Std***	***t***	***p***
Social condition	0.484	0.125	3.874	<0.001***
Correction condition	0.024	0.124	0.195	0.846
Social condition × Correction condition	-0.106	0.249	-0.428	0.669

Supplementary Table 36b. Perceived norm of responding predicted by social condition, correction condition, and interaction – ordinal logistic regression.

	b	***Std***	***t***	***p***
Social condition	0.214	0.127	1.688	0.091
Correction condition	0.299	0.127	2.351	0.019*
Social condition × Correction condition	-0.061	0.254	-0.241	0.809

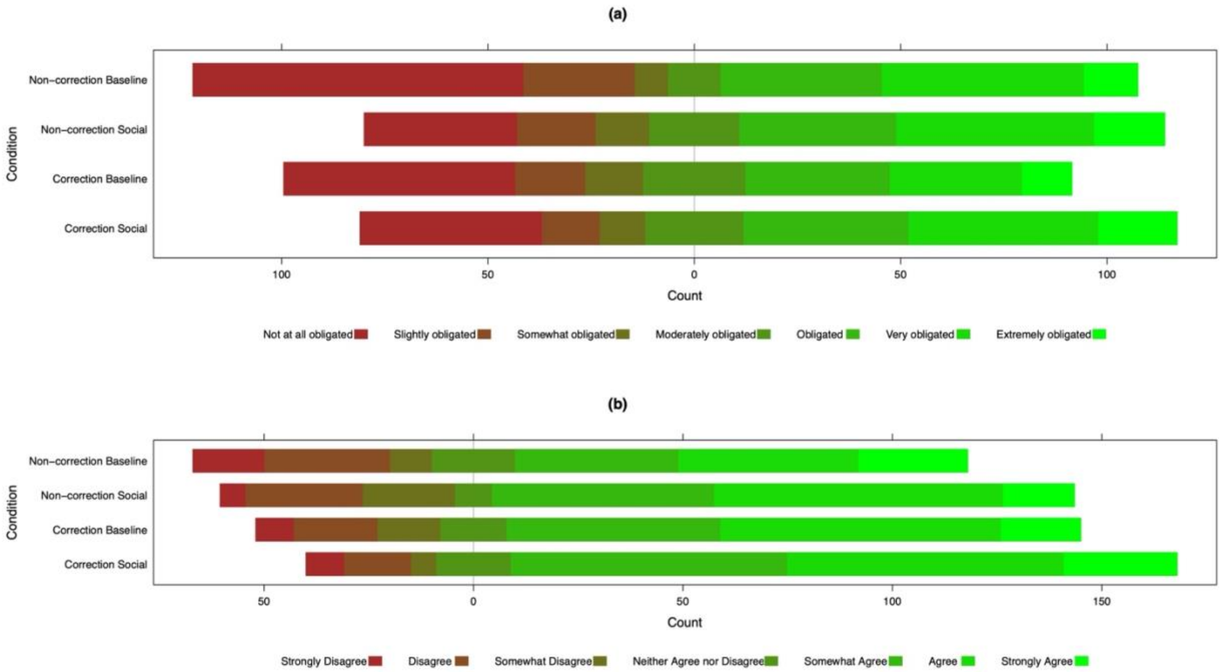

Supplementary Figure ***1****. Social connection increases reported personal obligation to reply and perceptions of a norm of reciprocal responding – diverging stacked bar charts displaying Likert-scale data.*

3. Supplemental Survey Experiment S1

a. Pre-registration.

We conducted a similar version of our main text survey experiment 1, reported here as Supplemental Survey Experiment S1 (N=1,290, M_{age} = 34.99, 420 female). This survey experiment was pre-registered (https://aspredicted.org/blind.php?x=OMI_DAZ) – full analyses and results are presented here as pre-registered. In this survey experiment, participants were first given a series of up to 24 possible true and false headlines (12 each). Upon sharing a false headline, participants advanced onto the main treatment part of the survey (N=948). Participants then received a fictitious response to their shared article (baseline, social x co-partisan, counter-partisan; concordance determined from corrector partisanship and Clinton vs. Trump binary choice item) and indicated whether they would reply. If participants indicated they would reply, they were then asked to write their free-response reply. This written response was then coded as either 'belief updating' or 'resisting information' by Amazon Mechanical Turk workers in a separate study (N=101 raters, each reply rated 10.56 times on average, Kendall's W=0.186, ICC(1,k)=0.570).

b. Descriptive Statistics.

Supplementary Table 41. Descriptive of engagements, belief updating responses, and resisting information responses by conditions (N=948) Belief updating and resisting information responses do not always equal total number of engagements, as some participants indicated they would engage, but did not write a free-response reply – as pre-registered, engagement was scored only using the binary choice reply item.

Experimental condition	*N*	Number of engagements	Number of positive responses	Number of negative responses
Baseline counter-partisan	248	116	61	40
Social counter-partisan	239	131	76	44
Baseline co-partisan	231	117	74	42
Social co-partisan	230	142	99	42

c. Main Analyses.

Supplementary Table 42. Effect of prior social connection and shared partisanship on engagement.

	b	***Std***	***t***	***p***
(Intercept)	0.537	0.016	33.216	<0.001***
Social condition	0.098	0.032	3.035	0.002**
Shared partisanship	0.050	0.032	1.533	0.126
Social condition X Shared partisanship	0.025	0.065	0.393	0.694

Supplementary Table 43. Effect of prior social connection and shared partisanship on belief updating.

	b	***Std***	***t***	***p***
(Intercept)	0.339	0.016	21.797	<0.001***
Social condition	0.093	0.031	2.983	0.003**
Shared partisanship	0.077	0.031	2.467	0.014*
Social condition X Shared partisanship	0.036	0.062	0.575	0.566

Supplementary Table 44. Effect of prior social connection and shared partisanship on resisting information.

	b	***Std***	***t***	***p***
(Intercept)	0.183	0.013	14.321	<0.001***
Social condition	0.012	0.026	0.467	0.641
Shared partisanship	-0.001	0.026	-0.038	0.970
Social condition X Shared partisanship	-0.022	0.051	-0.436	0.663

Supplementary Table 45. Significance of difference between coefficients of belief updating and resisting information.

	p
(Intercept)	<0.001***
Social condition	<0.001***
Shared partisanship	<0.001***
Social condition X Shared partisanship	0.099

Supplementary Table 46. Effect of prior social connection and shared partisanship on perceived positive corrector motive (1=corrector cares about truth; 0=corrector was politically motivated or trolling)..

	b	***Std***	***t***	***p***
(Intercept)	0.423	0.016	26.272	<0.001***
Social condition	0.020	0.033	0.623	0.533
Shared partisanship	0.084	0.033	2.574	0.010*
Social condition X Shared partisanship	0.059	0.059	0.901	0.368

d. Pre-registered Secondary Analyses.

Supplementary Table 47. Effect of prior social connection and shared partisanship on replying to argue with the corrector.

	**b**	**Std**	**t**	**p**
(Intercept)	0.085	0.009	9.362	<0.001***
Social condition	-0.008	0.018	-0.449	0.654
Shared partisanship	-0.014	0.018	-0.773	0.439
Social condition X Shared partisanship	0.034	0.036	0.944	0.345

Supplementary Table 48. Effect of prior social connection and shared partisanship on replying to discuss more with the corrector.

	**b**	**Std**	**t**	**p**
(Intercept)	0.179	0.012	14.465	<0.001***
Social condition	0.052	0.025	2.091	0.037*
Shared partisanship	0.058	0.025	2.336	0.020*
Social condition X Shared partisanship	0.002	0.050	0.045	0.964

Supplementary Table 49. Effect of prior social connection and shared partisanship on replying to thank the corrector.

	**b**	**Std**	**t**	**p**
(Intercept)	0.248	0.014	17.752	<0.001***
Social condition	0.063	0.028	2.271	0.023*
Shared partisanship	0.055	0.028	2.972	0.049*
Social condition X Shared partisanship	-0.029	0.056	-0.520	0.603

Supplementary Table 50. Effect of prior social connection and shared partisanship on not replying because participant knows they were correct.

	**b**	**Std**	**t**	**p**
(Intercept)	0.087	0.009	9.488	<0.001***
Social condition	0.004	0.018	0.222	0.825
Shared partisanship	-0.027	0.018	-1.465	0.143
Social condition X Shared partisanship	-0.008	0.037	-0.204	0.838

Supplementary Table 51. Effect of prior social connection and shared partisanship on not replying because participant knows corrector was correct.

	**b**	**Std**	**t**	**p**
(Intercept)	0.050	0.007	7.029	<0.001***
Social condition	-0.010	0.014	-0.712	0.476
Shared partisanship	0.000	0.014	0.034	0.973
Social condition X Shared partisanship	-0.040	0.028	-1.423	0.155

Supplementary Table 52. Effect of prior social connection and shared partisanship on not replying to avoid confrontation.

	**b**	**Std**	**t**	**p**

(Intercept)	0.201	0.013	15.402	<0.001***
Social condition	-0.027	0.026	-1.022	0.307
Shared partisanship	-0.029	0.026	-1.098	0.272
Social condition X Shared partisanship	0.038	0.052	0.720	0.472

Supplementary Table 53. Effect of prior social connection and shared partisanship on not replying because corrector was a stranger.

	b	***Std***	***t***	***p***
(Intercept)	0.122	0.011	11.512	<0.001***
Social condition	-0.065	0.021	-3.073	0.002**
Shared partisanship	0.011	0.021	0.536	0.592
Social condition X Shared partisanship	-0.016	0.042	-0.375	0.708

Supplementary Table 54. Effect of prior social connection and shared partisanship on self-reported belief updating.

	b	***Std***	***t***	***p***
(Intercept)	0.540	0.016	33.642	<0.001***
Social condition	0.025	0.032	0.793	0.428
Shared partisanship	0.130	0.032	4.049	<0.001***
Social condition X Shared partisanship	-0.124	0.064	-1.928	0.054

Supplementary Table 55. Effect of prior social connection and shared partisanship on self-reported resisting information.

	b	***Std***	***t***	***p***
(Intercept)	0.424	0.016	26.424	<0.001***
Social condition	-0.012	0.032	-0.387	0.699
Shared partisanship	-0.058	0.032	-1.820	0.069
Social condition X Shared partisanship	0.098	0.064	1.522	0.128

Supplementary Table 56. Effect of prior social connection, shared partisanship, and participant partisanship (z-scored; -0.5=Democrat, 0=Independent, 0.5=Republican) on engagement.

	b	***Std***	***t***	***p***
(Intercept)	0.524	0.017	31.719	<0.001***
Social condition	0.103	0.033	3.120	0.002**
Shared partisanship	0.076	0.033	2.293	0.022*
Participant partisanship	0.020	0.017	1.237	0.216
Social condition × Shared partisanship	-0.003	0.066	-0.047	0.963
Social condition × Participant partisanship	0.022	0.033	0.669	0.503
Shared partisanship × Participant partisanship	0.043	0.033	1.296	0.195

Social condition ✕ Shared partisanship ✕ Participant partisanship	-0.034	0.066	-0.517	0.605

Supplementary Table 57. Effect of prior social connection, shared partisanship, and participant partisanship (z-scored; -0.5=Democrat, 0=Independent, 0.5=Republican) on belief updating.

	b	***Std***	***t***	***p***
(Intercept)	0.342	0.016	21.686	<0.001***
Social condition	0.087	0.032	2.759	0.006**
Shared partisanship	0.073	0.032	2.322	0.020*
Participant partisanship	0.021	0.016	1.306	0.192
Social condition ✕ Shared partisanship	0.038	0.063	-0.606	0.545
Social condition ✕ Participant partisanship	0.006	0.032	0.178	0.859
Shared partisanship ✕ Participant partisanship	0.013	0.032	0.399	0.690
Social condition ✕ Shared partisanship ✕ Participant partisanship	-0.068	0.063	-1.084	0.279

Supplementary Table 58. Effect of prior social connection, shared partisanship, and participant partisanship (z-scored; -0.5=Democrat, 0=Independent, 0.5=Republican) on resisting information.

	b	***Std***	***t***	***p***
(Intercept)	0.179	0.013	13.939	<0.001***
Social condition	0.014	0.026	0.557	0.578
Shared partisanship	0.005	0.026	0.188	0.851
Participant partisanship	0.021	0.013	0.066	0.948
Social condition ✕ Shared partisanship	0.001	0.051	-0.727	0.467
Social condition ✕ Participant partisanship	-0.037	0.026	0.610	0.542
Shared partisanship ✕ Participant partisanship	0.033	0.026	1.300	0.194
Social condition ✕ Shared partisanship ✕ Participant partisanship	0.036	0.051	0.696	0.487

Supplementary Table 59. Effect of prior social connection and shared partisanship on engagement (where engagement = 0 if participant said they would reply, but did not actually write a free-response reply).

	b	***Std***	***t***	***p***
(Intercept)	0.508	0.016	31.482	<0.001***
Social condition	0.099	0.032	3.064	0.002**
Shared partisanship	0.105	0.032	3.260	0.001**
Social condition X Shared partisanship	0.011	0.065	0.175	0.861

e. Combined Survey Experiment 1 & Supplemental Survey Experiment S1: Exploratory Analysis

*Supplementary Table 60. Engagement predicted by social connection (centered), shared partisanship (centered), partisanship (binary; centered), and all interactions; with centered study-level dummy control. Data are combined from Main Text Survey Experiment 1 and Supplemental Survey Experiment S1. Linear hypothesis test comparing social condition and shared partisanship coefficients does not provide evidence of a statistically significant difference (*p*=.218).*

	b	*Std*	*t*	*p*
(Intercept)	0.545	0.012	45.422	<0.001***
Social condition	0.093	0.024	3.89	<0.001***
Shared partisanship	0.051	0.024	2.137	0.033*
Participant partisanship	-0.014	0.024	-0.583	0.56
Study	0.013	0.025	0.511	0.609
Social condition X Shared partisanship	0.055	0.048	1.151	0.25
Social condition X Participant partisanship	0	0.048	-0.005	0.996
Politics X Shared partisanship	-0.016	0.051	-0.314	0.754
Social condition X Shared partisanship X Participant partisanship	-0.009	0.095	-0.099	0.921

f. Bayesian Analyses.

Supplementary Table 61. Supplemental survey experiment S1 Bayesian regression analysis of effect of prior social connection, shared partisanship between participant and corrector, and their interaction on engagement using weakly-informative normal prior over intercept and effect of predictors and weakly-informative prior over residual standard deviation. l-95% CI shows the 0.025 percentile of posterior distribution and u-95% CI shows the 0.975 percentile of posterior distribution.

	Estimate	Estimate err.	l-95%	u-95%	$\hat{R}$	Bulk ESS	Tail ESS
(Intercept)	0.54	0.02	0.51	0.57	1	9123	5858
Shared partisanship	0.05	0.03	-0.01	0.11	1	11033	6283
Social condition	0.1	0.03	0.03	0.16	1	10892	6696
Shared partisanship X Social condition	0.03	0.06	-0.1	0.15	1	10345	6231

4. Twitter (X) Field Experiment

a. Materials.

Supplementary Table 62. Full list of 11 political articles rated false by fact-checking website Snopes.com. URLs used to find users who posted links to these articles on Twitter. Articles were selected via collecting the most recent political fact-checks written by Snopes.com in the two months prior to the Twitter field experiment (11-November-2019 to 3-January-2020). The average estimated partisanship of users who shared each fact-checked false claim is calculated using (2) on continuous interval [-2.5,2.5] where -2.5 represents strong liberal and 2.5 represents strong conservative.

	Claim	Fact-check URL	Averaged est. users' partisanship
1	Ukraine donated more money than any other country to the Clinton Foundation.	https://www.snopes.com/fact-check/ukraine-clinton-foundation/	1.61
2	In 2019, a U.S. District Court judge ruled that girls in an Illinois school	https://www.snopes.com/fact-check/girls-shower-with-boys-ruling/	1.19

	district "must shower with boys" and had no right to privacy.		
3	Donald Trump once evicted a disabled combat veteran for owning a small therapy dog.	https://www.snopes.com/fact-check/trump-veteran-service-dog/	-1.09
4	A photograph of U.S. President Donald Trump in his Trump Tower office in 2016 with several boxes of Sudafed in the background provides credible evidence of stimulant abuse.	https://www.snopes.com/fact-check/sudafed-trump-tower/	-0.79
5	The New York Times stated, as fact, that Hillary Clinton and George Soros had been responsible for paying a woman to make false allegations of sexual assault against Donald Trump.	https://www.snopes.com/fact-check/soros-clinton-trump-accuser/	1.71
6	An American diplomat named Melanie Honcharenko was found dead shortly before testifying in the impeachment inquiry against U.S. President Donald Trump.	https://www.snopes.com/fact-check/trump-impeachment-witness-die/	1.64
7	A proposed Virginia law would outlaw martial arts and firearms instruction.	https://www.snopes.com/fact-check/virginia-martial-arts-firearms-law/	1.72
8	"Illegal immigrants" killed 10,150 Americans in 2018.	https://www.snopes.com/fact-check/immigrant-homicides-us/	1.34
9	Virginia Gov. Ralph Northam said the National Guard would cut power and communications before killing anyone who didn't comply with new gun legislation.	https://www.snopes.com/fact-check/northam-meme/	1.74
10	A photograph shows Melania Trump with porn star Ron Jeremy.	https://www.snopes.com/fact-check/melania-trump-ron-jeremy-photo/	-0.26
11	Eric Trump tweeted about the airstrike that killed Iran Gen. Qassem Soleimani in early 2020 before the military operation took place.	https://www.snopes.com/fact-check/eric-trump-tweet-iran-strike/	-1.10

Supplementary Table 63. Full list of variations of the corrective messages used to debunk false content shared by users.

1	I don’t know if I believe this article - it might not be true I found a Snopes link that says this headline is false
2	I don’t know if I trust this article - it might not be true I found a Snopes link that says this headline is false
3	I'm not sure about the article you posted - it might not be true I found a link on Snopes that says this headline is false
4	I'm not sure about this article - it could be false I found a link on Snopes that says this headline is false
5	I'm not sure about this article - it may not be true I found a link on Snopes that says this headline is false
6	I'm not sure about this article - it might be false I found a link on Snopes that says this headline is false
7	I'm not sure about this article - it might be false I found a link on Snopes that says this headline is not true
8	I'm not sure about this article - it might not be true I found a link on Snopes that says this headline is false
9	I'm not sure about this article - it might not be true I found a link on Snopes that says this headline is not true
10	I'm not sure about this article - it might not be true I found a Snopes link that says this headline is false

11	I'm uncertain about this article - it might not be true I found a link on Snopes that says this headline is false
12	I'm unsure about this article - it might not be true I found a link on Snopes that says this headline is false

b. Descriptive Statistics.

Supplementary Table 64. Descriptive of engagements, positive responses, and negative responses by conditions (N=1,586).

Experimental condition	N_{Total}	$N_{DeliverySuccess}$	Number of engagements	Number of positive responses	Number of negative responses
Baseline counter-partisan	400	382	89	44	45
Social counter-partisan	401	329	89	42	47
Baseline co-partisan	396	381	90	44	46
Social co-partisan	389	364	110	47	63

c. Pre-registered Analyses.

In the pre-registration (https://aspredicted.org/F4C_3Y5), we specified our main analysis as a regression model predicting engagement by social condition (-0.5 = baseline, 0.5 = social), bot partisanship (i.e., -0.5=Democrat, 0.5=Republican), participant partisanship (Democrat versus Republican; z-score), and their interactions. In the main text we reported the results using shared partisanship between the bot and the user to be consistent with the analysis for the survey experiment. Here we report results using bot partisanship. These analyses also exclude users for whom treatment delivery was unsuccessful.

Supplementary Table 65. Effect of bot partisanship, prior social connection, and user partisanship on engagement.

	b	*Std*	*t*	p_{Reg}	p_{FRI}
(Intercept)	0.261	0.012	22.596	<0.001***	<0.001***
Social condition	0.051	0.023	2.221	0.027*	0.027*
Bot partisanship	-0.008	0.012	-0.665	0.504	0.496
User partisanship	0.001	0.012	0.086	0.946	0.8
Social condition ⨉ bot partisanship	0.019	0.023	0.837	0.420	0.4
Social condition ⨉ user partisanship	0.000	0.023	0.011	0.993	0.991
Bot partisanship ⨉ user partisanship	0.015	0.012	1.273	0.204	0.191
Social Condition ⨉ bot partisanship ⨉ user partisanship	0.006	0.023	0.297	0.780	0.744

Supplementary Table 66. Effect of bot partisanship, prior social connection, and user partisanship on positive engagement.

	b	*Std*	*t*	p_{Reg}	p_{FRI}
(Intercept)	0.122	0.009	14.167	<0.001***	<0.001***
Social condition	0.013	0.017	0.781	0.435	0.42
Bot partisanship	-0.013	0.009	-1.528	0.127	0.149
User partisanship	-0.017	0.009	-1.937	0.053	0.982
Social condition ⨉ bot partisanship	0.000	0.017	-0.009	0.993	0.99
Social condition ⨉ user partisanship	-0.005	0.017	-0.300	0.764	0.772
Bot partisanship ⨉ user partisanship	0.008	0.009	0.933	0.351	0.387
Social Condition ⨉ bot partisanship ⨉ user partisanship	0.001	0.017	0.051	0.960	0.961

Supplementary Table 67. Effect of bot partisanship, prior social connection, and user partisanship on negative engagement.

	b	***Std***	***t***	p_{Reg}	p_{FRI}
(Intercept)	0.139	0.009	15.301	<0.001***	<0.001***
Social condition	0.038	0.018	2.083	0.037*	0.034*
Bot partisanship	0.005	0.009	0.599	0.549	0.535
User partisanship	0.018	0.009	1.923	0.055	0.997
Social condition × bot partisanship	0.019	0.018	1.033	0.302	0.291
Social condition × user partisanship	0.005	0.018	0.296	0.767	0.743
Bot partisanship × user partisanship	0.007	0.009	0.733	0.463	0.437
Social Condition × bot partisanship × user partisanship	0.006	0.018	0.307	0.759	0.764

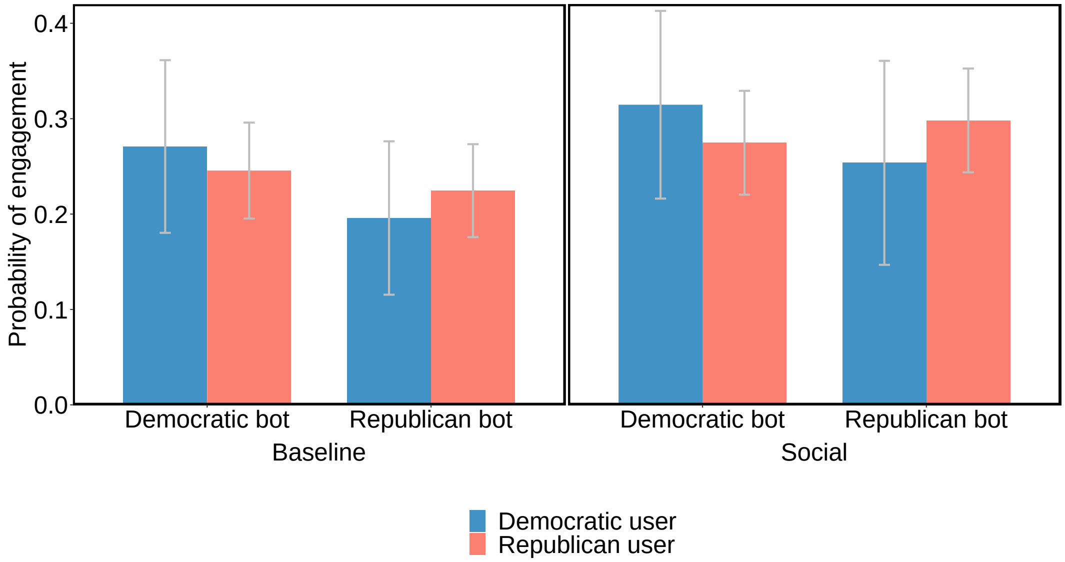

*Supplementary Figure **2**. Effect of bot partisanship, prior social connection, and user partisanship on engagement.*

Supplementary Table 68. Significance of difference between coefficients of positive engagement and negative engagement.

	p_{Reg}	p_{FRI}
(Intercept)	0.176	0.389
Social condition	0.335	0.349
Shared partisanship	0.137	0.155
User partisanship	0.006	0.157
Social condition × Bot partisanship	0.459	0.492
Social condition × User partisanship	0.686	0.680
Bot partisanship × User partisanship	0.920	0.890
Social Condition × Shared partisanship × User partisanship	0.861	0.876

d. Full Main Analyses – Messaged Users Only.

Supplementary Table 69. Effect of social connection on engagement among users in shared-partisanship condition (N=754).

	b	***Std***	***t***	***p***
(Intercept)	0.269	0.016	16.6	<0.001***
Social condition	0.066	0.032	2.03	0.042 *

Supplementary Table 70. Effect of social connection on negative engagement among users in shared-partisanship condition (N=754).

	b	***Std***	***t***	***p***
(Intercept)	0.147	0.013	11.4	<0.001***
Social condition	0.052	0.026	2.02	0.043*

Supplementary Table 71. Effect of social connection on positive engagement among users in shared-partisanship condition (N=754).

	b	***Std***	***t***	***p***
(Intercept)	0.122	0.012	10.2	<0.001***
Social condition	0.014	0.024	0.567	0.571

Supplementary Table 72. Effect of shared partisanship on engagement among users in baseline condition (N=763).

	b	***Std***	***t***	***p***
(Intercept)	0.236	0.022	10.9	<0.001***
Shared partisanship	0.003	0.031	0.105	0.916

Supplementary Table 73. Effect of shared partisanship on negative engagement among users in baseline condition (N=763).

	b	***Std***	***t***	***p***
(Intercept)	0.121	0.017	7.26	<0.001***
Shared partisanship	0.003	0.023	-0.125	0.901

Supplementary Table 74. Effect of shared partisanship on positive engagement among users in baseline condition (N=763).

	b	***Std***	***t***	***p***
(Intercept)	0.115	0.016	7.05	<0.001***
Shared partisanship	0	0.023	-0.013	0.99

Supplementary Table 75. Effect of shared partisanship, prior social connection, and user partisanship on engagement including users for whom correction was successful while observations are weighted by observations by each user's predicted probability of successful correction delivery had they been assigned to the social counter-partisan condition (N=1,456).

	b	***Std***	***t***	***p***
(Intercept)	0.255	0.011	22.3	<0.001***
Social condition	0.053	0.023	2.31	0.021*

Shared partisanship	0.007	0.011	0.571	0.568
User partisanship	-0.001	0.012	-0.068	0.946
Social condition ⨉ Shared partisanship	0.01	0.023	0.447	0.655
Social condition ⨉ User partisanship	0.004	0.024	0.164	0.87
Shared partisanship ⨉ User partisanship	-0.012	0.012	-1.05	0.295
Social Condition ⨉ Shared partisanship ⨉ User partisanship	0.009	0.024	0.397	0.691

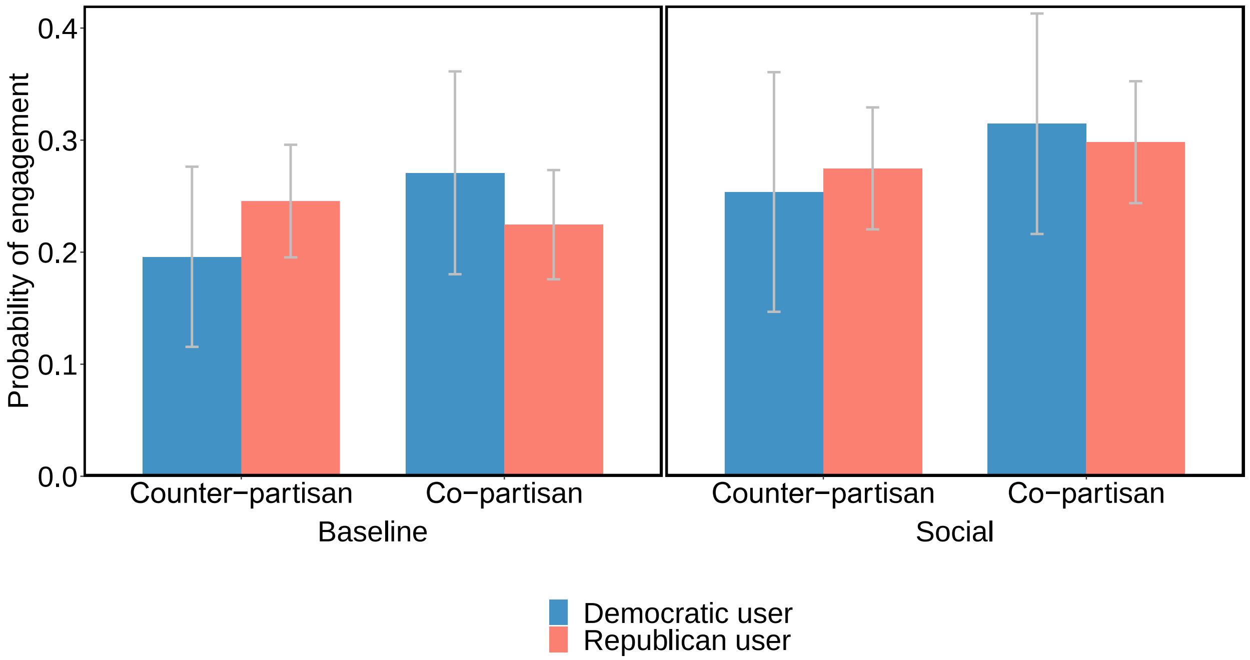

Supplementary Figure ***3****. Effect of shared partisanship, user partisanship, and prior social connection on probability of engagement.*

e. Bayesian Analyses.

Supplementary Table 76. Bayesian regression analysis of the effect of social connection on engagement among users in shared-partisanship condition (N=754) using weakly-informative normal prior over intercept and effect of predictors and weakly-informative prior over residual standard deviation. l-95% CI shows the 0.025 percentile of posterior distribution and u-95% CI shows the 0.975 percentile of posterior distribution.

	**Estimate**	**Estimate err.**	**l-95%**	**u-95%**	$\hat{R}$	**Bulk ESS**	**Tail ESS**
(Intercept)	0.27	0.02	0.24	0.3	1	9211	6276
Social connection	0.07	0.03	0	0.13	1	9354	5826

Supplementary Table 77. Bayesian regression analysis of the effect of social connection on negative engagement among users in shared-partisanship condition (N=754) using weakly-informative normal prior over intercept and effect of predictors and weakly-

informative prior over residual standard deviation. l-95% CI shows the 0.025 percentile of posterior distribution and u-95% CI shows the 0.975 percentile of posterior distribution.

	Estimate	Estimate err.	l-95%	u-95%	$\hat{R}$	Bulk ESS	Tail ESS
(Intercept)	0.15	0.01	0.12	0.17	1	8359	5981
Social connection	0.05	0.03	0	0.1	1	7911	5659

Supplementary Table 78. Bayesian regression analysis of the effect of social connection on positive engagement among users in shared-partisanship condition (N=754) using weakly-informative normal prior over intercept and effect of predictors and weakly-informative prior over residual standard deviation. l-95% CI shows the 0.025 percentile of posterior distribution and u-95% CI shows the 0.975 percentile of posterior distribution.

	Estimate	Estimate err.	l-95%	u-95%	$\hat{R}$	Bulk ESS	Tail ESS
(Intercept)	0.12	0.01	0.1	0.15	1	8035	5750
Social connection	0.01	0.02	-0.03	0.06	1	8303	6125

Supplementary Table 79. Bayesian regression analysis of effect of shared partisanship on engagement among users in the baseline condition using weakly-informative normal prior over intercept and effect of predictors and weakly-informative prior over residual standard deviation. l-95% CI shows the 0.025 percentile of posterior distribution and u-95% CI shows the 0.975 percentile of posterior distribution.

	Estimate	Estimate err.	l-95%	u-95%	$\hat{R}$	Bulk ESS	Tail ESS
(Intercept)	0.24	0.02	0.19	0.28	1	8193	6102
Shared partisanship	0.00	0.03	-0.06	0.06	1	8367	6462

Supplementary Table 80. Bayesian regression analysis of effect of shared partisanship on positive engagement among users in the baseline condition using weakly-informative normal prior over intercept and effect of predictors and weakly-informative prior over residual standard deviation. l-95% CI shows the 0.025 percentile of posterior distribution and u-95% CI shows the 0.975 percentile of posterior distribution.

	Estimate	Estimate err.	l-95%	u-95%	$\hat{R}$	Bulk ESS	Tail ESS
(Intercept)	0.12	0.02	0.08	0.15	1	8490	6198
Shared partisanship	0	0.02	-0.05	0.05	1	8174	6255

Supplementary Table 81. Bayesian regression analysis of effect of shared partisanship on negative engagement among users in the baseline condition using weakly-informative normal prior over intercept and effect of predictors and weakly-informative prior over residua standard deviation l. l-95% CI shows the 0.025 percentile of posterior distribution and u-95% CI shows the 0.975 percentile of posterior distribution.

	Estimate	Estimate err.	l-95%	u-95%	$\hat{R}$	Bulk ESS	Tail ESS
(Intercept)	0.12	0.02	0.09	0.15	1	7718	5826
Shared partisanship	0	0.02	-0.05	0.04	1	6956	5950

Supplementary Table 82. Bayesian regression analysis of effect of shared partisanship, prior social connection, and user partisanship on engagement including users for whom correction was successful (N=1,456) while observations are weighted by observations by each user's predicted probability of successful correction delivery had they been assigned to the social counter-partisan condition. Results are generated using weakly-informative normal prior over intercept and effect of predictors and

weakly-informative prior over residual standard deviation. l-95% CI shows the 0.025 percentile of posterior distribution and u-95% CI shows the 0.975 percentile of posterior distribution.

	Estimate	Estimate err.	l-95%	u-95%	$\hat{R}$	Bulk ESS	Tail ESS
(Intercept)	0.26	0.01	0.23	0.28	1	13822	6427
Social condition	0.05	0.02	0	0.1	1	12350	6081
Shared partisanship	0.01	0.01	-0.02	0.03	1	12748	5856
User partisanship	0	0.01	-0.03	0.02	1	12220	6437
Social condition × Shared partisanship	0.01	0.02	-0.04	0.06	1	12449	5823
Social condition × User partisanship	0	0.03	-0.05	0.05	1	12650	5912
Shared partisanship × User partisanship	-0.01	0.01	-0.04	0.01	1	13637	6369
Social Condition × Shared partisanship × User partisanship	0.01	0.03	-0.04	0.06	1	12672	5708

1.5 Article-level and corrective message level analysis

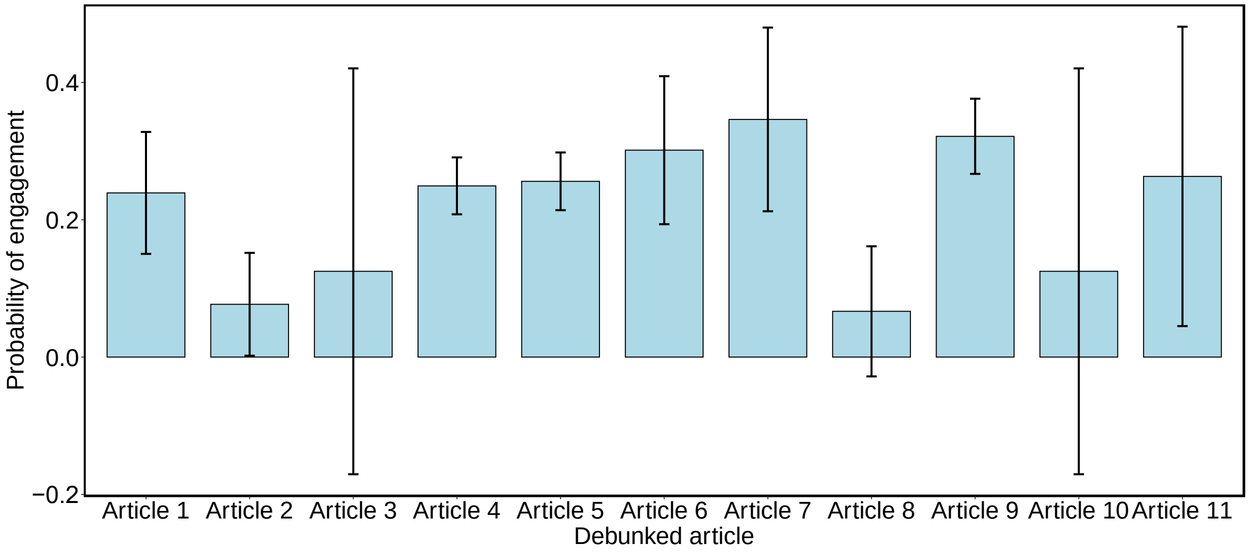

Supplementary Figure ***4****. Probability of user engagement with corrective message for each debunked article. The number of observations per each article is not sufficient to draw meaningful statistical inference in terms of heterogeneous effect of the article on probability of engagement. Article numbers correspond to Supplementary Table 1.*

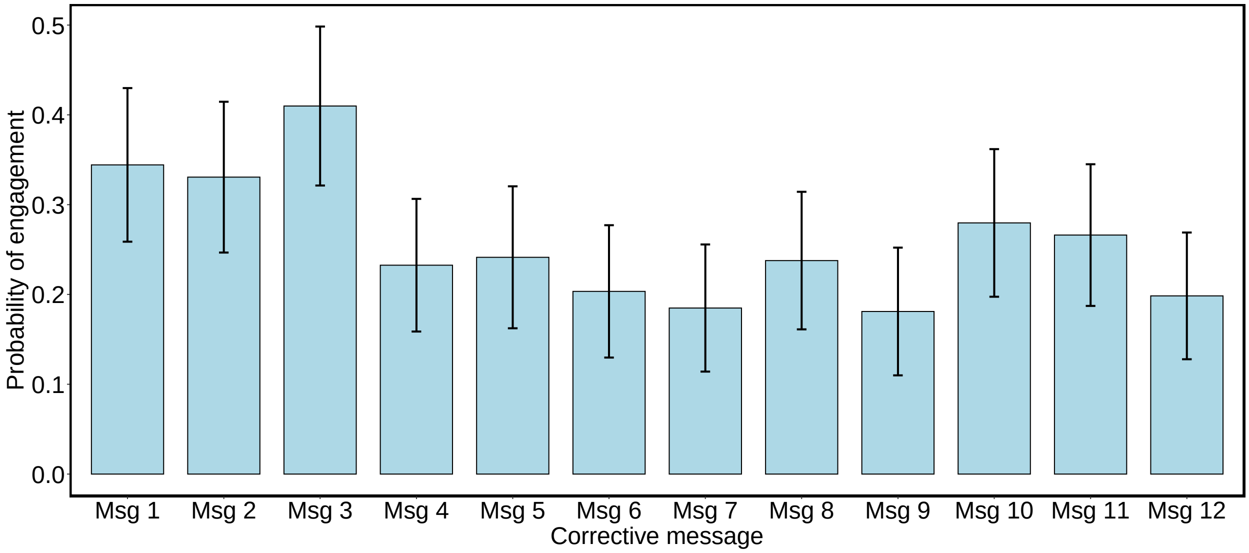

*Supplementary Figure **5**. Probability of user engagement with corrective message for each correction message variation. Corrective message wordings were not fully randomized as part of our experimental procedure. Message numbers correspond to Supplementary Table 2.*

f. Reply Tweet Rating.

In order to assess whether reply tweets in the field experiment were receptive or rejecting of our corrective messages, we recruited 66 Amazon Mechanical Turk workers to each evaluate 50 unique reply tweets and rate them as either 'belief updating' or 'resisting information.' Raters were told they would be asked to read a number of tweets, where in each case the writer had first shared a news article on Twitter, and then had received a response message indicating that the news article was false. Raters were also told that the correction message included a real link to a Snopes article. Raters then were instructed that they would be reading the replies Twitter users then wrote to such correction messages. For each reply, raters were asked to categorize the reply as either 'belief updating' or 'resisting information,' defined as follows (motivated by (3)):

Belief Updating: Replies that indicate that the replier originally thought the article they shared was true, but after seeing the correction message now believe that what they shared is false. Belief updating can be thought of as the correction successfully changing the mind of the replier, such that they now know they shared fake news. These replies may involve saying **thank you**, saying **sorry**, or indicating that they will **delete their original post.**

Example - "Thank you for your message, I guess I was wrong and the article I posted was fake."
Example - "Thanks, sorry I usually fact check my posts better. I will delete my Tweet."

Resisting Information: Replies that argue that their original post is true, argue that the correction itself is false or misleading, or argue in support of the general idea or sentiment behind the original post being true. Generally speaking, any attempt to counter-argue or ignore the correction is an example of resisting information. Replies that do not engage with the correction and simply indicate some level of ignorance or apathy towards the original poster also constitute resisting information. These replies may involve saying that the **correction is fake**, arguing that **there is information refuting the correction**, or **ignoring the correction** while making other arguments or claims.

Example - "I think the article I posted is correct, because I've read it on other news sites."
Example - "Snopes is fake news."
Example - "I don't know what to believe anymore."

Raters were also instructed that all Twitter handles had been anonymized to "@username," but that other than this modification, all tweets were presented as they actually appeared on Twitter.

If a user in the field experiment replied to a correction message with multiple tweets, these tweets were combined into a single unique tweet reply for rating purposes.

For each reply tweet, raters first read the tweet, and then were asked: "Does this reply belong to the category of **belief updating** (e.g., "Thanks, sorry, I usually fact check my posts better. I will delete by Tweet.") or **resisting information** (e.g., "I think the article I posted is correct, because I've read it on other news sites. Also, Snopes is fake news.")? (*Belief Updating*; *Resisting Information*).

For each reply tweet, the aggregate reply tweet type was recorded as the modal rating. Deleted tweets and favourited or retweeted correction messages (if there was no written reply) were also recorded as types of positive engagement. Resisting information was recorded as negative engagement.

Supplementary Table 83. Different types of tweet reactions coded as positive engagement.

Types of Positive Engagement

Modal rater evaluation of written reply indicated response expressed **belief updating** (as defined above)	Original tweet containing false news was deleted by user after successful correction delivery	User favourited or retweeted the correction message (conditional on there being no written reply)
136	13	28

g. Supplementary Results Text.

4g.1. Positive and negative engagement - principal stratification analysis.

For positive engagement, we did not find a significant effect of social condition (b=0.014, p=0.413), nor of shared partisanship (b=-0.001, p=0.915), and did not find interaction between conditions (b=0.002, p=0.902). For negative engagement, we found a significant effect of social condition (b=0.038, p=0.038), did not find a significant effect of shared partisanship (b=0.009, p=0.306), and did not observe an interaction between conditions (b=0.014, p=0.443). We also did not find a significant difference in the effect of social connection on negative versus positive engagement (p= 0.385); though this null result should be interpreted with caution, as it is plausible that social connection may still differentially increase negative engagement more so than positive engagement, as our null effects were not precisely estimated.

4g.2. Field experiment analyses including all users.

We conducted several additional analyses with the goal of incorporating data from the social counter-partisan condition, despite differential treatment delivery failure. First, including users for whom message delivery failed, we do not find evidence of an effect of social condition, shared partisanship, nor their interaction (ps>.125; Figure S5a,b), nor an effect on positive engagement or negative engagement (ps>.138). These results suggest that at the bot campaign level, social connection did not increase correction engagement if you specify that those who never received the correction did not engage with the correction – however, it may be the case that those who never received the correction could have been more likely to engage with the correction conditional on receiving it, and thus it is difficult to assess how these results apply to actual engagement with corrective messages.

4g.3. Field experiment analyses excluding users for whom message delivery failed.

When excluding users for whom message delivery failed, we find a significant positive effect of prior social connection on correction engagement (b=0.052, SE=0.023, t(1455)= 2.242 , p_{reg}=0.025, p_{FRI}=.017; Figure S5c,d). We also did not find a significant effect of shared partisanship on engaging with the correction (b=0.009, SE=0.012, t(1455)= 0.748, p_{reg}=0.454, p_{FRI}=0.435; 95% confidence interval on null effect [-0.01, 0.03]), nor did we find a significant interaction between prior social connection and shared partisanship (b=0.016, SE=0.023, t(1455)=0.690, p_{reg}=0.490, p_{FRI}=0.475).

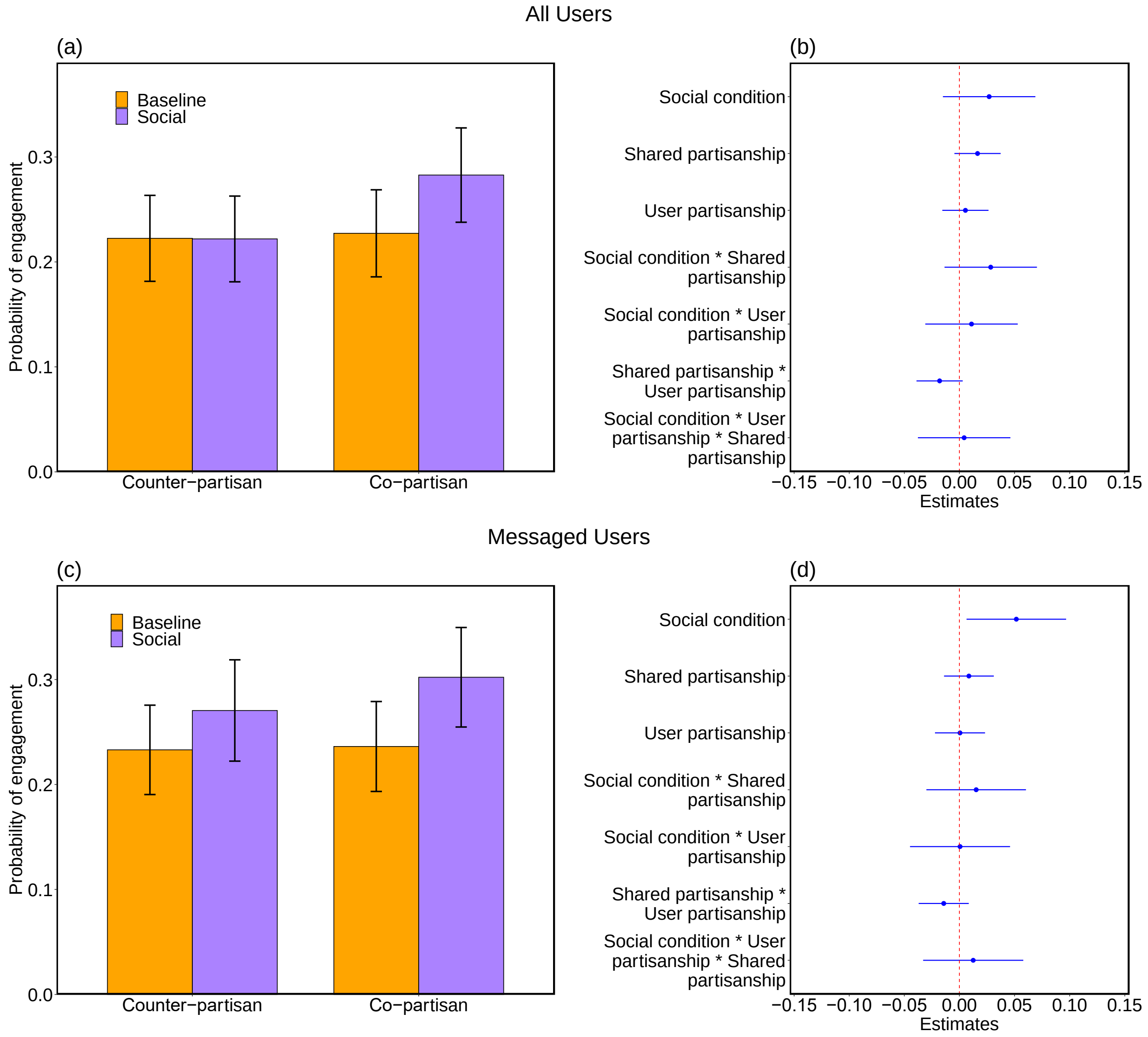

Supplementary Figure 6. Prior social connection increases engagement with corrections in our Twitter field experiment when assessing messaged users. Probability of engagement with the corrective message in the field experiment is shown across experimental conditions. Panels (a) and (b) show, respectively, the probability of engagement and effect sizes from the linear model when including users for whom the correction message failed to send. Panels (c) and (d) show, respectively, the probability of engagement and effect sizes from the linear model when excluding users for whom the correction message failed to send. Error bars reflect 95% confidence intervals.

4g.4. Positive and negative engagement analyses excluding users for whom message delivery failed.

For positive engagement, we do not find a significant effect of prior social connection (b=0.013, SE=0.017, t(1455)=0.782, p_{reg}=0.435, p_{FRI}=0.430), shared partisanship (b=0.000, SE=0.009, t(1455)=0.006, p_{reg}=0.995, p_{FRI}=0.996), or their interaction (b=0.001, SE=0.017, t(1455)=0.039, p_{reg}=0.955, p_{FRI}=0.961). For negative engagement, we also do not find a significant effect of shared partisanship (b=0.009, SE=0.009, t(1455)=0.945, p_{reg}=0.345, p_{FRI}=0.303), and do not find a significant interaction between shared partisanship and prior social connection (b=0.015, SE=0.018, t(1455)=0.824, p_{reg}=0.410, p_{FRI}=0.410). We do, however, find a significant main effect of social connection on negative engagement (b=0.038, SE=0.018, t(1455)=2.111, p_{reg}=0.035, p_{FRI}=0.033). Of note, the effect of prior social connection on negative versus positive engagement was not significantly different (p_{reg}=0.325, p_{FRI}=0.395). As in our analyses examining the effect of social connection amongst the co-partisan conditions only, we again do not find evidence that the social condition increased negative engagement *more* than it increased positive engagement (for full results, see Supplementary Tables 9-11, 13-14). However, we again emphasize

that this absence of significant difference between negative and positive engagement should be interpreted with caution, as we still find a directionally stronger effect of social connection increasing negative engagement more so than positive engagement.

We also again find some evidence of a partisan asymmetry in engagement valence. User partisanship is not associated with overall engagement (b=0.001, SE=0.012, $t(1455)$=0.064, p_{reg}=0.949), and we do not observe any significant interactions between user partisanship and either of our experimental conditions (ps>0.075). However, we do find that more conservative users were marginally less likely to engage positively with the correction (b=-0.017, SE=0.009, $t(1455)$=-1.975, p_{reg}=0.050), and were also marginally more likely to engage negatively with the correction (b=0.018, SE=0.009, $t(1455)$=1.944, p_{reg}=0.052) - such that more conservative users were more likely to express negative than positive engagement (p_{reg}=0.006). These results provide preliminary evidence of a partisan asymmetry in responses to corrective information, although it is important to keep in mind that in terms of partisanship our sample is not representative of either Twitter users or Americans more generally.

h. Follow-back Analyses.

To verify users were likely attending to bot partisanship, we conduct an analysis of the relative follow-back rates of users for co-partisan and counter-partisan bots, with the expectation that if users attended to partisanship, then follow-back rates would be higher for co-partisan than counter-partisan connections (see (4)).

Supplementary Table 84. Effect of shared partisanship and user partisanship on probability of follow-back in social condition.

	b	***Std***	***T***	***p***
(Intercept)	0.174	0.016	10.780	<0.001***
Shared partisanship	0.196	0.032	6.086	<0.001***
User partisanship	0.129	0.032	3.837	<0.001***
Shared partisanship × User partisanship	-0.005	0.064	-0.078	0.938

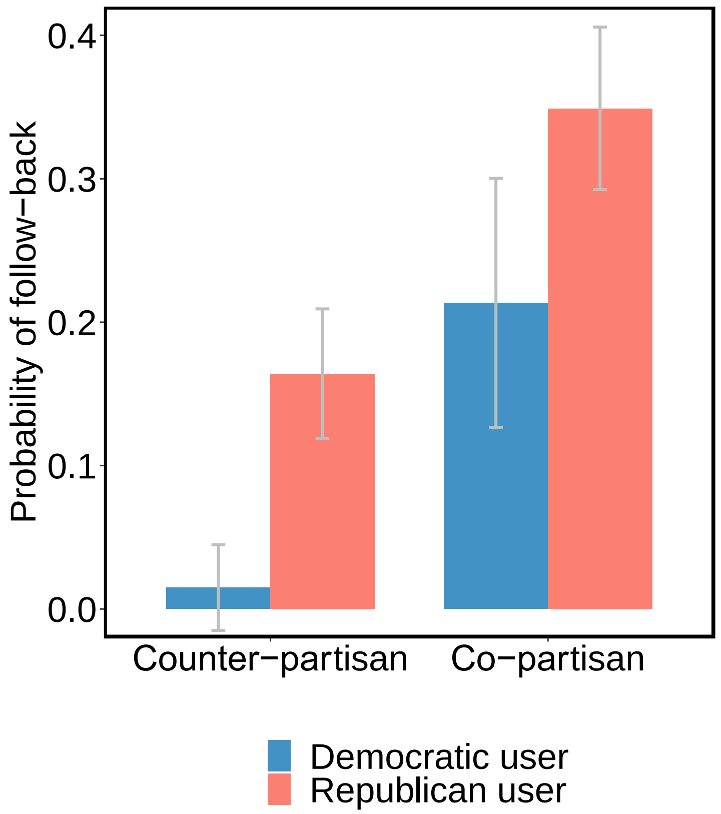

Supplementary Figure 7. Effect of shared partisanship and user partisanship on probability of follow-back in social condition.

Supplementary Table 85. Effect of bot partisanship and user partisanship on probability of follow-back in social condition.

	b	*Std*	*t*	*p*
(Intercept)	0.224	0.016	10.780	<0.001***
Bot partisanship	-0.003	0.032	-0.078	0.938
User partisanship	0.129	0.032	3.993	<0.001***
Bot partisanship × user partisanship	0.391	0.064	6.069	<0.001***

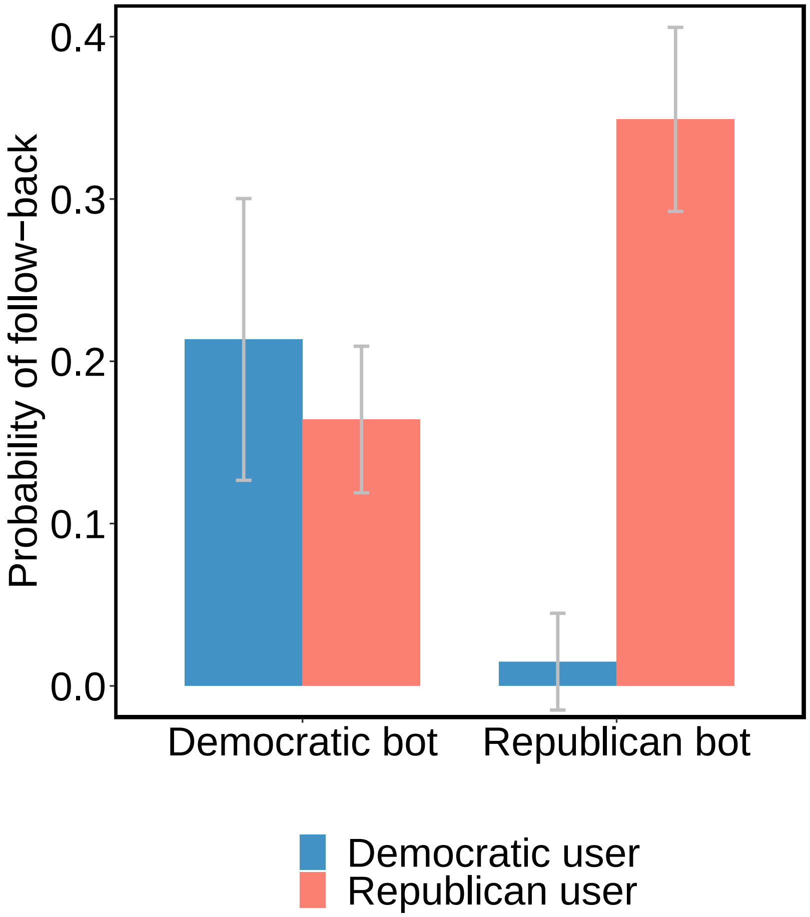

Supplementary Figure 8. Effect of bot partisanship and user partisanship on probability of follow-back in social condition.

5. Partisan Extremity Analyses: Field Experiment & Main Survey Experiment 1

The following exploratory analyses examine the role of partisan extremity (absolute value of *z*-scored partisanship) predicting correction engagement across social connection and shared partisanship conditions.

a. Relationship between Partisan Extremity and Correction Delivery Failure (i.e., Blocking) in Field Experiment.

Supplementary Table 86. Correction delivery failure (i.e., blocking) predicted by user partisan extremity (absolute value of estimated partisanship) in Social, Counter-partisan field experiment condition.

	b	***Std***	***t***	***p***
(Intercept)	0.035	0.061	0.570	0.569
Social condition	0.093	0.038	2.460	0.014*

Supplementary Table 87. User partisan extremity by field experiment condition

**Baseline, Counter-partisan**	**Baseline, Co-partisan**	**Social, Counter-partisan**	**Social, Co-partisan**
Extremity: 1.53	Extremity: 1.52	Extremity: 1.53	Extremity: 1.52

Supplementary Table 88. ANOVA predicting user partisan extremity by field experiment condition.

	df	***Mean sq.***	***F***	***p***
Condition	3	0.042	0.16	0.920
Residuals	1452	0.263	---	---

b. User Partisan Extremity in Field Experiment.

Supplementary Table 89. Effect of shared partisanship, prior social connection, and user partisan extremity on engagement (N=1,456).

	b	***Std***	***t***	***p***
(Intercept)	0.261	0.011	22.796	<0.001***
Social condition	0.053	0.023	2.305	0.021*
Shared partisanship	0.008	0.011	0.710	0.478
User partisan extremity	0.049	0.011	4.269	<0.001***
Social condition ⨉ Shared partisanship	0.013	0.023	0.547	0.584
Social condition ⨉ User partisan extremity	0.021	0.023	0.921	0.357
Shared partisanship ⨉ User partisan extremity	-0.008	0.011	-0.684	0.494
Social Condition ⨉ Shared partisanship ⨉ User partisan extremity	0.017	0.023	0.755	0.451

Supplementary Table 90. Effect of shared partisanship, prior social connection, and their interaction on correction engagement for lowest 3 bins of user partisan extremity (N=1,115).

	b	***Std***	***t***	***p***
(Intercept)	0.245	0.013	19.05	<0.001***
Social condition	0.055	0.026	2.14	0.033*
Shared partisanship	0.007	0.013	0.54	0.588
Social condition X Shared partisanship	-0.018	0.026	-0.71	0.477

Supplementary Table 91. Effect of shared partisanship, prior social connection, and their interaction on correction engagement for highest bin of user partisan extremity (N=335).

	b	***Std***	***t***	***p***
(Intercept)	0.312	0.025	12.34	<0.001***
Social condition	0.046	0.051	0.91	0.360
Shared partisanship	0.016	0.025	0.63	0.529
Social condition X Shared partisanship	0.118	0.051	2.33	0.020*

Supplementary Table 92. Effect of shared partisanship, prior social connection, and user partisan extremity dummy (0=lower 3 partisan extremity bins, 1=greatest partisan extremity bin) on engagement (N=1,456).

	b	***Std***	***t***	***p***
(Intercept)	0.245	0.013	18.705	<0.001***
Social condition	0.055	0.026	2.094	0.036*
Shared partisanship	0.007	0.013	0.532	0.595
User partisan extremity	0.067	0.027	2.461	0.014*
Social condition ⨯ Shared partisanship	-0.018	0.026	-0.698	0.485
Social condition ⨯ User partisan extremity	-0.010	0.054	-0.187	0.852
Shared partisanship ⨯ User partisan extremity	0.009	0.027	0.329	0.742
Social Condition ⨯ Shared partisanship ⨯ User partisan extremity	0.136	0.054	2.501	0.012*

Supplementary Table 93. Effect of shared partisanship, prior social connection, and user partisan extremity dummy (0=lower 3 partisan extremity bins, 1=greatest partisan extremity bin) on engagement for all users (N=1,586).

	b	***Std***	***t***	***p***
(Intercept)	0.238	0.011	22.378	<0.001***
Social condition	0.028	0.021	1.327	0.185
Shared partisanship	0.017	0.011	1.593	0.111
User partisan extremity	0.027	0.011	2.494	0.013*
Social condition ⨯ Shared partisanship	0.028	0.021	1.318	0.188
Social condition ⨯ User partisan extremity	-0.007	0.021	-0.315	0.753
Shared partisanship ⨯ User partisan extremity	0.007	0.011	0.690	0.490
Social Condition ⨯ Shared partisanship ⨯ User partisan extremity	0.064	0.021	2.994	0.003**

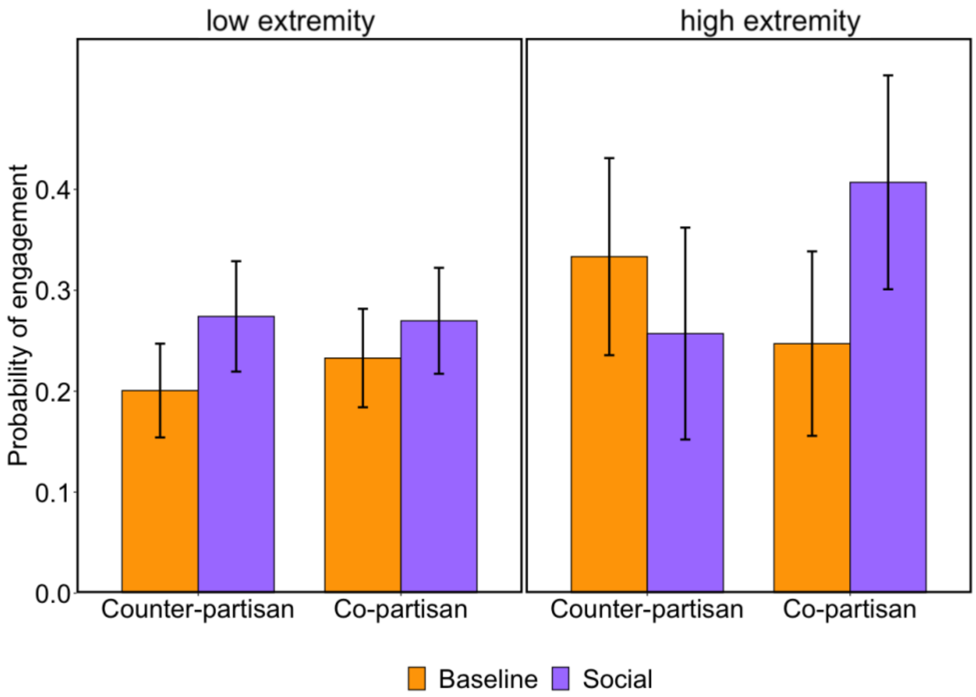

Supplementary Figure 9. Effect of prior social connection and shared partisanship on correction engagement for all users, by user partisan extremity (binned: low extremity = lowest 3 bins, high extremity = highest bin). Error bars reflect 95% confidence intervals.

c. Participant Partisan Extremity in Main Survey Experiment 1

Supplementary Table 94. Effect of shared partisanship, prior social connection, and participant partisan extremity on engagement (N=808).

	b	***Std***	***t***	***p***
(Intercept)	0.553	0.017	31.821	<0.001***
Social condition	0.089	0.035	2.559	0.011*
Shared partisanship	0.027	0.017	1.545	0.123
User partisan extremity	0.048	0.017	2.733	0.006**
Social condition ⨉ Shared partisanship	0.048	0.035	1.389	0.165
Social condition ⨉ User partisan extremity	0.001	0.035	0.30	0.976
Shared partisanship ⨉ User partisan extremity	0.015	0.017	0.854	0.394
Social Condition ⨉ Shared partisanship ⨉ User partisan extremity	0.017	0.035	0.476	0.634

Supplementary Table 95. Effect of shared partisanship, prior social connection, and their interaction on correction engagement for lowest 3 bins of participant partisan extremity (N=586).

	b	***Std***	***t***	***p***
(Intercept)	0.549	0.021	26.806	<0.001***
Social condition	0.101	0.041	2.460	0.014*
Shared partisanship	0.023	0.021	1.109	0.268
Social condition X Shared partisanship	-0.013	0.041	-0.315	0.753

Supplementary Table 96. Effect of shared partisanship, prior social connection, and their interaction on correction engagement for highest bin of user partisan extremity (N=222).

	b	***Std***	***t***	***p***
(Intercept)	0.564	0.033	17.200	<0.001***
Social condition	0.046	0.066	0.703	0.483
Shared partisanship	0.042	0.033	1.283	0.201
Social condition X Shared partisanship	0.201	0.066	3.062	0.002**

Supplementary Table 97. Effect of shared partisanship, prior social connection, and user partisan extremity dummy (0=lower 3 partisan extremity bins, 1=greatest partisan extremity bin) on engagement (N=808).

	b	***Std***	***t***	***p***
(Intercept)	0.549	0.020	26.924	<0.001***
Social condition	0.101	0.041	2.471	0.014*
Shared partisanship	0.023	0.020	1.114	0.267
User partisan extremity	0.014	0.039	0.370	0.711
Social condition × Shared partisanship	-0.013	0.041	-0.316	0.752
Social condition × User partisan extremity	-0.055	0.078	-0.703	0.483
Shared partisanship × User partisan extremity	0.019	0.039	0.496	0.620
Social Condition × Shared partisanship × User partisan extremity	0.214	0.078	2.743	0.006*

6. References

1. J. Jordan, E. Yoeli, D. Rand, Don’t get it or don’t spread it? Comparing self-interested versus prosocially framed COVID-19 prevention messaging. *PsyArXiv* **10** (2020).
2. P. Barberá, J. T. Jost, J. Nagler, J. A. Tucker, R. Bonneau, Tweeting from left to right: Is online political communication more than an echo chamber? *Psychological Science* **26**, 1531-1542 (2015).
3. M. Prasad *et al.*, “There must be a reason”: Osama, Saddam, and inferred justification. *Sociological Inquiry* **79**, 142-162 (2009).
4. M. Mosleh, C. Martel, D. Eckles, D. G. Rand, Shared partisanship dramatically increases social tie formation in a Twitter field experiment. *Proceedings of the National Academy of Sciences* **118** (2021).
5. K. Munger, Tweetment effects on the tweeted: Experimentally reducing racist harassment. *Political Behavior* **39**, 629-649 (2017).
6. D. Freelon, M. Bossetta, C. Wells, J. Lukito, Y. Xia, K. Adams, Black trolls matter: Racial and ideological asymmetries in social media disinformation. *Social Science Computer Review* **0894439320914853** (2020).
7. A. Friggeri, L. Adamic, D. Eckles, J. Cheng, Rumor cascades. *Proceedings of the International AAI Conference on Web and Social Media.* (2014).
8. J. Shin, L. Jian, K. Driscoll, F. Bar, Political rumoring on Twitter during the 2012 US presidential election: Rumor diffusion and correction. *New Media & Society* **19**, 1214-1235 (2017).
9. M. Mosleh, C. Martel, D. Eckles, D. G. Rand, Perverse Downstream Consequences of Debunking: Being Corrected by Another User for Posting False Political News Increases Subsequent Sharing of Low Quality,

Partisan, and Toxic Content in a Twitter Field Experiment. *Proceedings of the 2021 CHI Conference on Human Factors in Computing Systems* (2021).
10. L. Bode, E. K. Vraga, See something, say something: Correction of global health misinformation on social media. *Health Communication* **33**, 1121-1140.
