# Supplementary material for: Promoting engagement with social fact-checks online: Investigating the roles of social connection and shared partisanship
Source: PLoS One. 2025 Mar 31;20(3):e0319336. doi: 10.1371/journal.pone.0319336 (PMC11957346; doi:10.1371/journal.pone.0319336)
Supplement: Attachment — Submitted filename: Response to Reviewers.pdf [file pone.0319336.s002.pdf]

January 8, 2025

Dear Dr. Cresci,

Thank you very much for the opportunity to submit a revised version of our manuscript entitled "Promoting engagement with social fact-checks online: Investigating the roles of social connection and shared partisanship" We are thankful that the reviewers thought our manuscript is "compelling" and "of general interest for social media researchers and experts."

We also want to thank the reviewers for their useful insights and suggestions. We have addressed all reviewer comments in full. Broadly, we (1) further describe and justify the methodological and analytic decisions made across our experiments, (2) focus our paper framing and discussion more concretely on the factors of shared partisanship and minimal social connection, (3) expanded our discussion on limitations and constrains on generality, offering additional directions for future research, and (4) have more clearly structured and documented our code and replication materials available on this project's OSF page. Below we provide detailed point-by-point responses to all of the comments.

We believe that the manuscript has substantially improved, and hope that you will find the revised version suitable for publication in *PLOS ONE*.

Thank you again for your work on our manuscript,

David Rand

<u>Editor comments</u>

1) If you would like to make changes to your financial disclosure, please include your updated statement in your cover letter. Guidelines for resubmitting your figure files are available below the reviewer comments at the end of this letter.

We have revised our funding statement / financial disclosure as follows.

"The authors gratefully acknowledge funding from Google through the Google Research 611 Scholar Award, gifts from Google and Meta to support other research, and funding via 612 the National Science Foundation Graduate Research Fellowship under Grant No. 613 174530. The funders had no role in study design, data collection and analysis, decision 614 to publish, or preparation of the manuscript." (p. 15)

2) Both reviewers found merit in this manuscript, however both also identified a number of relevant issues that must be addressed for the paper to be published. Among the issues requiring particular attention are the methodological choices which require better justification and description, as well as improvements in certain areas; and some aspects of the study design that need stronger justification or the adoption of alternative approaches to avoid possible biases.

We greatly appreciate the reviewers encouraging us to better explain our analytic and procedural decisions when conducting our experiments and analyses. We have revised the manuscript to better justify, describe, and improve upon these choices.

Namely, we have (1) clarified our analytic choices for using linear models, Bayesian regression analyses robustness checks for null results, and a random forest model for treatment delivery classification as part of our principal stratification approach; (2) revised the text to better clarify the binning procedures for our exploratory extreme partisan analyses; (3) added additional robustness checks and graphical representations of our Likert-scale data from survey experiment 2 being treated as ordinal rather than interval; (4) clarified and justified our operationalization of minimal social relationships in our field experiment; and (5) justify and explain our decisions to correct users who shared the most recently fact-checked false headlines and use candidate (dis)preference (Trump) as our main proxy for partisan identification when manipulating shared partisanship. On this latter point, we emphasize that these decisions do not introduce bias into our causal estimation of the effect of shared partisanship – rather, they result in a majority of our sample being Republican leaning in our field experiment (which is more representative of misinformation sharers in the U.S. context) and reflect partisan preferences at the time of our study (Republicans overwhelmingly supported Trump; Democrats overwhelmingly were opposed to Trump).

For convenience, we have reproduced our relevant responses to Reviewers 1 and 2 on these points below.

We have revised our manuscript to clarify the reasoning behind our analytic choices. Namely, (a) linear models were pre-registered as our main analyses and are the primary analyses reported throughout the text, given OLS's robustness, flexibility, and high level of interpretability; (b) exploratory Bayesian regression analyses were conducted and reported as additional robustness checks around the estimation of null hypotheses to add more clarity as to the precision of these null effects; and (c) a random forest model was conducted as part of our principal stratification approach for correcting our differential treatment delivery failure rates since random forests are a well-documented high accuracy and efficiency classifier model, and our principal stratification approach relies on accurately predicting the probability of correction treatment delivery by user characteristics.

"…we find via a linear probability model (as pre-registered; linear models used as primary analyses in the current work unless stated otherwise)…" (p. 5)

"…using Bayesian regression analysis [throughout the current work, we perform exploratory Bayesian regression analyses to provide additional robustness checks around our precise estimation of null hypotheses]…" (p. 5)

"We chose to use a random forest classifier for this prediction task given research demonstrating random forest classification can be a highly accurate and effective classification procedure [36] – and the better we can predict successful intervention delivery for users, the better our principal

stratification approach can help address differential message delivery failure rates across conditions." (p. 6)

We also better explain the binning process and analyses for our exploratory extreme partisanship analyses. Participants in our survey experiment are binned such that individuals who reported extreme scale points of partisanship (1 or 7 on our 7-point partisanship measure) are characterized as extreme partisans (as they selected the most extreme partisanship options). About 28% of our participants are extreme partisans in our survey sample as per this metric. As a best proxy for this in our field data, we created bins of users from the absolute value of their inferred partisanship score (0 to 2.5), and classified the highest bin (1.88 to 2.5) as extreme partisans, as this also roughly translated to selecting the highest quarter of extreme partisans using an interval binning similar to our Likert binning in our survey experiment. That is, an inferred score of 1.88 to 2.5 reflects very strong partisanship as per the partisan inference metric from Barberá et al. (2015), and selecting a 1 or 7 scale extreme point for partisanship reflects strong partisanship on our partisan Likert scale.

We do also want to emphasize that these analyses are purely exploratory and are not pre-registered – we conducted them to further try and examine how social connection and shared partisanship may be interrelated beyond our pre-registered analyses.

We have revised the text to clarify these above points.

"However, we also investigated whether treatment effects may have differed for the most politically extreme users in our sample (i.e., 1 or 7 on 7-point partisanship measure; N=222, 27.5% of total sample). In an exploratory, non-pre-registered analysis…" (p. 9)

"To match our survey experiment analyses, we classified users into four bins based on partisan extremity (binned by inferred absolute value of partisanship [25] intervals of 0.625, from 0 to 2.5) and compared the most extreme users (highest bin: partisan score 1.88 to 2.5) to all other users (N=355 high political extremity, 24.4% of total sample) in order to again compare the roughly quarter most politically extreme users to all other users." (pp. 9-10)

"These results provide suggestive evidence that for less politically extreme users, social connection is beneficial overall, whereas for more politically extreme users, social connection is only beneficial among co-partisans – though we again note that these analyses examining high versus low politically extreme users are purely exploratory." (p. 10)

Reviewer 2 also raised a point about treating our Likert-scale outcome variables as ordinal rather than interval. We contend (and pre-registered) that our scales of obligated responding and perceptions of a norm of reciprocal responding should best be interpreted as interval, with equidistance between our scale points – and thus we report the results of our linear model analyses in the main text. And accordingly, in Figure 5 we plot the mean and 95% confidence intervals of this continuous outcome by social and correction conditions. This graphical representation also helps provide visual synergy in demonstrating the causal

effects of social condition here as compared to in our field experiment and survey experiment 1.

However, we appreciate Reviewer 2's suggestion of treating these data as ordinal and plotting these data using a diverging stacked bar chart. As a robustness check, we fit ordinal logistic regression models in addition to our pre-registered linear models for survey experiment 2. Consistent with our primary results, we find that social connection increases reported personal obligation to reply (b=0.484, p<.001) and nominally increases perceptions of a norm of reciprocal responding (b=0.214, p=.091) as assessed via ordinal logistic regression models. These results are reported in full in SI Tables 35b and 36b. We also plot these data using diverging stacked bar charts (from the HH package as suggested) – these visualizations are available in SI Figure S1.

We also clarify in our text and response to reviewers' letter several of our methodological design choices.

Reviewer 1 encouraged us to more clearly outline how we operationalized minimal social connections in our experiment. In our field experiment methods, we clarify that the key component of our social connection is establishing a unilateral tie between the corrector and to-be-corrected accounts. Liking three recent tweets was added to this condition in order to increase perceived authenticity and the likelihood that targeted users saw and remembered our accounts having initiated social contact.

"We also varied whether each account followed and liked the three most recent tweets of the to-be-corrected users one day prior to sending correction replies – thus establishing a minimal social connection. The primary component of this social connection is the unilateral tie created by our following the targeted account. Liking three recent tweets was included as part of this treatment in order to increase the perceived authenticity of our accounts, as well as increase the likelihood that users saw and remembered notifications from our account." (p. 4)

Given the time and resources necessary for conducting a field experiment of this nature, we decided not to further manipulate the extent of this minimal social relationship (i.e., liking fewer or more tweets; commenting on other posts; etc.). To address this limitation on generality, we also revised the manuscript to suggest that future work may investigate how varying such prior social contact might further impact engagement with social corrections.

"Our experiments are also limited by our selection and operationalization of minimal social connection and shared partisanship as corrector features of interest for our studies. First when establishing minimal social connections, in addition to initiating a unilateral social tie by following users on Twitter (X), we also liked three of users' tweets from the previous day in order to increase the salience and verisimilitude of our accounts. Future work may examine whether the extent of this minimal social contact may further impact the probability of users subsequently responding to corrective messages – for instance, liking a greater or lesser number of previous posts, or initiating more extensive contact such as commenting on other posts before issuing a corrective message." (pp. 13-14)

Reviewer 2 commented on our sampling procedure of which users to correct based on recently fact-checked false articles, as well as on our design of partisan corrector accounts in our field experiment.

We clarify in our response to Reviewer 2 that in our current work, we focus on examining the causal effects of shared partisanship and social connection – we do not highlight in our results or discussion partisan differences, given the asymmetries noted in the false articles selected for and in the design of our partisan accounts (as described in comment 4 below). That is, it is true that we corrected majority estimated conservative users in our field experiment (75.4%, as reported in our main text) and that the average estimate of users' partisanship who shared the majority of the fact-checked false URLs we selected for leaned more conservative than liberal. But this does not bias our causal estimates of the effects of shared partisanship or minimal social connections – all users in our sample were still randomly assigned to either shared or opposing partisanship, and minimal or no minimal social connection. Thus, the causal estimates of these treatments are not biased by our selection of users from these 11 selected false articles – rather, this just dictates that our sample consists of majority Republican users.

Further, an abundance of research suggests that if anything, this sampling decision actually makes our sample more representative of the actual population of online users sharing misinformation. Multiple papers (Baribi-Bartov et al., 2024 in *Science*; González-Bailón et al., 2023 in *Science*; Grinberg et al., 2019 in *Science*; Guess et al., 2019 in *Science Advances*; Mosleh et al., 2024 in *Nature*) provide evidence that the majority of misinformation / articles from low-quality news domains are shared by political conservatives. Thus, rather than forcing an artificial partisanship balance, our procedure of sampling by the most recently fact-checked false political articles likely produces a more actually representative sample of users who were sharing false news at the time of our field experiment.

And regarding our design of partisan accounts as indicating partisanship via 'Trump 2020' or 'Anyone but Trump 2020' affiliation, we clarify in our response that this decision only introduces potential bias when interpreting the non-causal association between partisanship and correction engagement – *not* when examining the causal effect of shared partisanship on correction engagement, since shared partisanship is randomly assigned and aggregated across all users. We focus on this latter causal effect of shared partisanship as our key manipulation in the current work (as pre-registered).

We also note that our field experiment was conducted before there was a presumptive 2020 Democratic Party Presidential nominee, and thus 'Anyone but Trump 2020' was the best Democratic equivalent to 'Trump 2020' that could be implemented at the time.

And as further assurance, there was incredibly high alignment between favor for or against Trump and political party at the time of our field experiment (November 2019). According to Gallup polling (https://news.gallup.com/poll/203198/presidential-approval-ratings-donald-trump.aspx), November 2019 Presidential approval for Trump was 90% amongst Republicans and 4% amongst Democrats. Indicating Trump support versus opposition highly aligned with political party at the time of our field experiment – which is precisely

how we then chose to operationalize partisanship in order to manipulate shared partisanship in our study.

That said, it is interesting to consider how other operationalizations of partisanship may impact the effect of shared partisanship on correction engagement. We note this, as well as our above points, in our revised discussion section to better clarify these points and future directions for readers.

"Second, given the time in which we conducted our field experiment, we operationalized account partisanship primarily through candidate preference. Given that candidate preference at the time we began our field experiment was highly indicative of partisanship [50], we posit that this is an effective way to manipulate shared or misaligned partisan preferences. That said, further research may examine how different representations expressing partisan preference (e.g., different candidate preferences; statements about party or issue position preferences) may differentially impact perceived shared partisanship and subsequent correction engagement." (p. 14)

3) A reviewer also commented that the provided supplementary materials and code were insufficiently documented and unclear.

We greatly appreciate Reviewer 2 raising this issue. We have revised and reuploaded our replication files on OSF accordingly. Our response to Reviewer 2, comment 9 is reproduced below for convenience.

First, as suggested, we have added a README file to better guide interested readers through different folders and files. Additionally, we have renamed files to more intuitively match their relation to the main text and supporting information documents. We believe this README file and our renamed and reorganized OSF documents will greatly improve navigation and understanding of our data and analysis code.

Second, we have gone through our analysis files and more clearly documented discrete analysis steps and procedures, as well as highlight which analyses are pre-registered and which are exploratory. As now noted in the README, the order of analyses in each analysis file is now the order they appear in the codebook.

Third, we have fixed the code in the file "SnopeFieldReplies.Rmd" that required manual updating of computed NA values. The initial code was structured to compute the proportion of raters who evaluated a given field correction reply as demonstrating "resisting information" – but the initial code improperly dealt with NAs for cases of unanimous reporting (hence why NAs were previously manually fixed depending on which way the unanimous rating classified the post as evident from the response coding data – either 100% resisting information or 100% belief updating). We have fixed this bug and updated our code such that no manual NA recoding is required (see lines 45-48 in R markdown file) – all classifications remain identical as initially coded.

Fourth, we have removed the use of absolute file paths requiring "setwd" – we now use the "here" library and here() function to set the file path to the current working directory, and have set up our OSF files such that each analysis file shares the same directory as the data files it requires. This is also specified now in our README file.

Journal requirements

(1) When submitting your revision, we need you to address these additional requirements. Please ensure that your manuscript meets PLOS ONE's style requirements, including those for file naming. The PLOS ONE style templates can be found at https://journals.plos.org/plosone/s/file?id=wjVg/PLOSOne_formatting_sample_main_body.pdf and https://journals.plos.org/plosone/s/file?id=ba62/PLOSOne_formatting_sample_title_authors_affiliations.pdf.

We have revised our files to meet PLOS ONE's style requirements and proper file naming procedure.

(2) Please update your submission to use the PLOS LaTeX template. The template and more information on our requirements for LaTeX submissions can be found at http://journals.plos.org/plosone/s/latex.

We have updated our submission to use the PLOS LaTeX template.

(3) Thank you for stating the following financial disclosure: [The authors gratefully acknowledge funding from Google through Google Research Scholar Award, gifts from Google to support other research, and funding via the National Science Foundation Graduate Research Fellowship under Grant No. 174530.]. Please state what role the funders took in the study. If the funders had no role, please state: ""The funders had no role in study design, data collection and analysis, decision to publish, or preparation of the manuscript."" If this statement is not correct you must amend it as needed. Please include this amended Role of Funder statement in your cover letter; we will change the online submission form on your behalf.

We have revised our funding statement / financial disclosure as follows.

"The authors gratefully acknowledge funding from Google through the Google Research Scholar Award, gifts from Google and Meta to support other research, and funding via the National Science Foundation Graduate Research Fellowship under Grant No. 174530. The funders had no role in study design, data collection and analysis, decision to publish, or preparation of the manuscript." (p. 15)

(4) Thank you for stating the following in the Competing Interests section: [Other research by the authors was funded by Meta and Google.]. Please confirm that this does not alter your adherence to all PLOS ONE policies on sharing data and materials, by including the following statement: ""This does not alter our adherence to PLOS ONE policies on sharing data and materials." (as detailed online in our guide for authors http://journals.plos.org/plosone/s/competing-interests). If there are restrictions on sharing of data and/or materials, please state these. Please note that we cannot proceed with consideration of your article until this information has been declared. Please include your

updated Competing Interests statement in your cover letter; we will change the online submission form on your behalf.

Confirmed – we have updated our Competing Interests statement accordingly.

"Other research by the authors is funded by Meta and Google. This does not alter our adherence to PLOS ONE policies on sharing data and materials." (p. 15)

(5) Thank you for stating the following in the Acknowledgments Section of your manuscript: [Authors thank Ben Tappin for insightful comments on Bayesian analysis and Antonio Arechar for assistance with survey experiment data collection. The authors gratefully acknowledge funding from Google through Google Research Scholar Award, gifts from Google to support other research, and funding via the National Science Foundation Graduate Research Fellowship under Grant No. 174530.] We note that you have provided funding information that is not currently declared in your Funding Statement. However, funding information should not appear in the Acknowledgments section or other areas of your manuscript. We will only publish funding information present in the Funding Statement section of the online submission form. Please remove any funding-related text from the manuscript and let us know how you would like to update your Funding Statement. Currently, your Funding Statement reads as follows: [The authors gratefully acknowledge funding from Google through Google Research Scholar Award, gifts from Google to support other research, and funding via the National Science Foundation Graduate Research Fellowship under Grant No. 174530.]. Please include your amended statements within your cover letter; we will change the online submission form on your behalf.

We have removed funding information from our Acknowledgements and updated our Acknowledgements Section and Funding Statement accordingly.

They are as follows.

"Acknowledgments
Authors thank Ben Tappin for insightful comments on Bayesian analysis and Antonio 608 Arechar for assistance with survey experiment data collection." (p. 15)

"Funding Statement
The authors gratefully acknowledge funding from Google through the Google Research Scholar Award, gifts from Google and Meta to support other research, and funding via the National Science Foundation Graduate Research Fellowship under Grant No. 174530. The funders had no role in study design, data collection and analysis, decision to publish, or preparation of the manuscript." (p. 15)

(6) We note that you have indicated that there are restrictions to data sharing for this study. PLOS only allows data to be available upon request if there are legal or ethical restrictions on sharing data publicly. For more information on unacceptable data access restrictions, please see http://journals.plos.org/plosone/s/data-availability#loc-unacceptable-data-

access-restrictions. Before we proceed with your manuscript, please address the following prompts: a) If there are ethical or legal restrictions on sharing a de-identified data set, please explain them in detail (e.g., data contain potentially identifying or sensitive patient information, data are owned by a third-party organization, etc.) and who has imposed them (e.g., a Research Ethics Committee or Institutional Review Board, etc.). Please also provide contact information for a data access committee, ethics committee, or other institutional body to which data requests may be sent. b) If there are no restrictions, please upload the minimal anonymized data set necessary to replicate your study findings to a stable, public repository and provide us with the relevant URLs, DOIs, or accession numbers. For a list of recommended repositories, please see https://journals.plos.org/plosone/s/recommended-repositories. You also have the option of uploading the data as Supporting Information files, but we would recommend depositing data directly to a data repository if possible. We will update your Data Availability statement on your behalf to reflect the information you provide.

We have uploaded a minimal anonymized data set necessary to replicate our field study findings to OSF here: https://osf.io/nvk4u/.

(7) Please include captions for your Supporting Information files at the end of your manuscript, and update any in-text citations to match accordingly. Please see our Supporting Information guidelines for more information: http://journals.plos.org/plosone/s/supporting-information.

We have included a caption for our Supporting Information file at the end of our manuscript.

"S1 Supporting Information. Supporting information file. Supporting information for "Promoting engagement with social fact-checks online: Investigating the roles of social connection and shared partisanship"" (p. 14)

We have also updated all in-text citations to match accordingly, in the format of "Table X in S1 Supporting Information" as directed in the Supporting Information guidelines.
For example, see below.

"…see Table 62 in S1 Supporting Information for list of stories…" (p. 3)

<u>Reviewer 1 comments</u>

(1) The authors report that the data will be available although "some restrictions will apply." These restrictions are not outlined.

In our original manuscript, these restrictions referred to the Twitter (X) data being only available upon request for confidentiality reasons. However, we have revised our data availability such that a minimal anonymized field data set is available to replicate our primary study findings (OSF data file name: 'SnopeReplies_Complete.csv'; OSF analysis file name; Field_Analysis.Rmd). Thus, all anonymized data are now available with no restrictions. We have revised our manuscript and data sharing disclosures accordingly. Our OSF project is here: https://osf.io/nvk4u/?view_only=2ba8efe34cd647e18f69857bd1f85bbd.

"All analysis code and minimal anonymized data required for replicating our primary field analyses are available here." (p. 4)

(2) Page 3: "Our experiments focus on two factors which prior work suggest may be important for eliciting engagement with corrections: partisan alignment between the corrector and the corrected user, and social connection between the corrector and the corrected user." -- are there other factors the authors think would be relevant to explore in future research aside from these 2? For example, one could study whether the correction post came from a highly educated individual or not, or if the correction post was engaged with (liked, retweeted) or not (and if so, by whom).

We thank Reviewer 1 for these interesting additional factors which should certainly be explored in future research on correction engagement. In our current work, we focus on partisan alignment and social connection, given that prior literature has focused on these two factors but primarily consists of either only correlational or survey-based research – and here, we extend this by conducting our field experiment and additional survey experiments. However, we agree that additional factors such as education and third-party correction engagement are important topics for further work. We have revised our discussion to speak to these future directions.

"And finally, in the current work we focus on the corrector factors of partisan alignment and social connection between correctors and corrected users. Future work should examine whether other corrector or correction traits (e.g., if the correction receives likes or retweets from other third-party users) or relationships between correctors and corrected users (e.g., education level similarity or disparity between correctors and corrected users) may additionally impact correction engagement." (p. 14)

(3) Page 4: "What characteristics of correction senders promote engagement with social corrections?" -- is this meant to be the research question motivating this study? This sentence does not flow well with the paragraph that follows given that the authors are studying 2 very specific characteristics (partisanship and social connection). In other words, the authors aren't answering the question they pose.

We have revised the manuscript to more clearly describe our primary question of interest.

"What are the effects of partisan alignment and virtual social connection on engagement with corrections? Here, we experimentally test this question." (pp. 2-3)

(4) Page 4: "We varied whether our corrector bots identified as Democrats or Republicans, and whether they followed the user and liked three of their tweets the previous day (thus creating a minimal social relationship)." -- did the authors come up with this definition of "minimal social relationship"? Why 3 likes instead of 2 or 10? This seems arbitrary, and is not justified in the current version of the manuscript. This plays a rather large role in the framing/design of the studies and needs to be clarified.

We thank Reviewer 1 for encouraging us to more clearly outline how we operationalized minimal social connections in our experiment. In our field experiment methods, we clarify that the key component of our social connection is establishing a unilateral tie between the corrector and to-be-corrected accounts. Liking three recent tweets was added to this condition in order to increase perceived authenticity and the likelihood that targeted users saw and remembered our accounts having initiated social contact.

"We also varied whether each account followed and liked the three most recent tweets of the to-be-corrected users one day prior to sending correction replies – thus establishing a minimal social connection. The primary component of this social connection is the unilateral tie created by our following the targeted account. Liking three recent tweets was included as part of this treatment in order to increase the perceived authenticity of our accounts, as well as increase the likelihood that users saw and remembered notifications from our account." (p. 4)

Given the time and resources necessary for conducting a field experiment of this nature, we decided not to further manipulate the extent of this minimal social relationship (i.e., liking fewer or more tweets; commenting on other posts; etc.). To address this limitation on generality, we also revised the manuscript to suggest that future work may investigate how varying such prior social contact might further impact engagement with social corrections.

"Our experiments are also limited by our selection and operationalization of minimal social connection and shared partisanship as corrector features of interest for our studies. First when establishing minimal social connections, in addition to initiating a unilateral social tie by following users on Twitter (X), we also liked three of users' tweets from the previous day in order to increase the salience and verisimilitude of our accounts. Future work may examine whether the extent of this minimal social contact my further impact the probability of users subsequently responding to corrective messages – for instance, liking a greater or lesser number of previous posts, or initiating more extensive contact such as commenting on other posts before issuing a corrective message. (pp. 13-14)

(5) For the field experiment, how was the Twitter data collected?

All Twitter data were collected using the (no longer existing) Academic Twitter API. We have revised the manuscript to address this question.

"All Twitter (X) field data were collected using the (then existing) Academic Twitter API." (p. 5)

(6) Footnote 1 on page 6: The information there is highly redundant as the 2 deviations are also reported on page 5. Suggest removing redundant content.

Agreed – we have removed the redundant footnote, and our deviations are now just reported in the text.

(7) The methods of analysis are not clearly motivated or explained anywhere in the paper. For example, the authors use a random forest model on Page 7. Why? The reader is never brought through the methodological choice. Bayesian regression and GLMs are used at other points in the study. Again, why? These methodological components should be clearly introduced before being deployed.

We have revised our manuscript to clarify the reasoning behind our methodological choices. Namely, (a) linear models were pre-registered as our main analyses and are the primary analyses reported throughout the text, given OLS's robustness, flexibility, and high level of interpretability; (b) exploratory Bayesian regression analyses were conducted and reported as additional robustness checks around the estimation of null hypotheses to add more clarity as to the precision of these null effects; and (c) a random forest model was conducted as part of our principal stratification approach for correcting our differential treatment delivery failure rates since random forests are a well-documented high accuracy and efficiency classifier model, and our principal stratification approach relies on accurately predicting the probability of correction treatment delivery by user characteristics.

"…we find via a linear probability model (as pre-registered; linear models used as primary analyses in the current work unless stated otherwise)…" (p. 5)

"…using Bayesian regression analysis [throughout the current work, we perform exploratory Bayesian regression analyses to provide additional robustness checks around our precise estimation of null hypotheses]…" (p. 5)

"We chose to use a random forest classifier for this prediction task given research demonstrating random forest classification can be a highly accurate and effective classification procedure [36] – and the better we can predict successful intervention delivery for users, the better our principal stratification approach can help address differential message delivery failure rates across conditions." (p. 6)

Reviewer 2 comments

(1) Regarding the framing of the study, I believe that the title and abstract should be more specific with respect to the work carried out. For instance, in the abstract it is stated that "we ask what types of social correction promote engagement from corrected users", which initially led me to think that the study focuses on variations of the correction themselves. However, the study only focuses on two traits between corrector and corrected users: partisanship and online social tie, with the latter being narrowed down to the corrector following the corrected user, i.e., a unidirectional and unilateral tie.

We have revised our title and abstract to better clarify the focus of our current work.

"Promoting engagement with social fact-checks online: Investigating the roles of social connection and shared partisanship" (p. 1)

"Here we ask under what conditions social corrections promote engagement from corrected users, allowing for greater insight into how users respond to debunking messages (even if such responses are negative). Prior work suggests two key factors may help promote engagement with corrections – partisan alignment between users, and social connections between users. We investigate these factors here." (p. 1)

(2) In addition, I think the sociogeographic context of the study (the US political ecosystem a few months prior to the 2020 US elections) should be clearly stated and described, as this might have influenced the results and might not be obvious for readers not familiar with US politics. Hence, I believe the discussion and conclusions should be framed more narrowly.

We have added a section in our discussion and conclusions section on this sociographic context of our current work.

"And fourth, our field experiment and survey experiment 1 were conducted within the year leading up to the 2020 U.S. Presidential election. Thus, our results reflect the specific sociographic context of this time and location – the lead-up to a presidential contest within a polarized two-party system. Contemporary work may examine whether similar effects of social connection and shared partisan are observed under different political climates, cross-nationally, and in multi-party systems." (p. 13)

(3) Regarding the construction of the fact-checking dataset and bot accounts for the field experiment, I found some balance issues that might have affected results. In particular, there is —in my view— a noticeable imbalance in the estimated liberal–conservative partisanship scale (measured from -2.5 to +2.5) of the eleven fact-check URLs of Snopes —§4.a of the Supplemental Information (SI). As can be seen on Table 62 of SI, seven of the eleven Snopes' URLs have a marked conservative estimated partisanship (ranging from +1.19 to +1.74), whereas the remaining four liberal URLs are more center leaning (ranging from -0.27 to -1.1). Perhaps, instead of taking the absolute most recent articles, it would have been better to filter them until obtaining a better partisanship balance.

We thank Reviewer 2 for raising this point. In our current work, we focus on examining the causal effects of shared partisanship and social connection – we do not highlight in our results or discussion partisan differences, given the asymmetries noted in the false articles selected for and in the design of our partisan accounts (as described in comment 4 below). That is, it is true that we corrected majority estimated conservative users in our field experiment (75.4%, as reported in our main text) and that the average estimate of users' partisanship who shared the majority of the fact-checked false URLs we selected for leaned more conservative than liberal. But this does not bias our causal estimates of the effects of shared partisanship or minimal social connections – all users in our sample were still randomly assigned to either shared or opposing partisanship, and minimal or no minimal social connection. Thus, the causal estimates of these treatments are not biased by our selection of users from these 11 selected false articles – rather, this just dictates that our sample consists of majority Republican users.

And second, an abundance of research suggests that if anything, this sampling decision actually makes our sample more representative of the actual population of online users sharing misinformation. Multiple papers (citations below) provide evidence that the majority of misinformation / articles from low-quality news domains are shared by political conservatives. Thus, rather than forcing an artificial partisanship balance, our procedure of sampling by the most recently fact-checked false political articles likely produces a more actually representative sample of users who were sharing false news at the time of our field experiment.

Baribi-Bartov, S., Swire-Thompson, B., & Grinberg, N. (2024). Supersharers of fake news on Twitter. *Science*, *384*(6699), 979-982.

González-Bailón, S., Lazer, D., Barberá, P., Zhang, M., Allcott, H., Brown, T., ... & Tucker, J. A. (2023). Asymmetric ideological segregation in exposure to political news on Facebook. *Science*, *381*(6656), 392-398.

Grinberg, N., Joseph, K., Friedland, L., Swire-Thompson, B., & Lazer, D. (2019). Fake news on Twitter during the 2016 US presidential election. *Science*, *363*(6425), 374-378.

Guess, A., Nagler, J., & Tucker, J. (2019). Less than you think: Prevalence and predictors of fake news dissemination on Facebook. *Science Advances*, *5*(1), eaau4586.

Mosleh, M., Yang, Q., Zaman, T., Pennycook, G., & Rand, D. G. (2024). Differences in misinformation sharing can lead to politically asymmetric sanctions. *Nature*, 1-8.

We have revised our discussion to mention that our sample is non-representative of the general American public – but does reflect, as suggested by the cited empirical work above, the population of users more likely to share false and low-quality content in the U.S. context.

"Third, Mechanical Turk samples and Twitter (X) users who post links to debunked articles are highly non-representative of the American public. For instance, the majority of users in our field experiment are politically right-leaning – which, while non-representative of the general public, is reflective of empirical work showing that political conservatives are more likely to share and be exposed to low-quality content online [45–49]." (p. 13)

(4) In addition, in the Procedure paragraph of Survey Experiment 1, it is stated that "corrector partisanship was indicated by candidate preference in the fictional account Twitter name", with the caption of Figure 2.b indicating "Democrat condition left; Republican condition right". However, the respective usernames read "Anyone but Trump 2020" and "Trump 2020", which I would argue it's only reflective of partisanship in favor or against Trump, but not necessarily towards a political party. In my view, the respective username statements are not equivalent and might have introduced bias against the so-called Democrat condition.

First regarding bias, this again only introduces potential bias when interpreting the non-causal association between partisanship and correction engagement – not when examining the causal effect of shared partisanship on correction engagement, since shared partisanship is randomly assigned and aggregated across all users.

Second, our field experiment was conducted before there was a presumptive 2020 Democratic Party Presidential nominee, and thus 'Anyone but Trump 2020' was the best Democratic equivalent to 'Trump 2020' that could be implemented at the time.

And third, there was incredibly high alignment between favor for or against Trump and political party at the time of our field experiment (November 2019). According to Gallup polling (https://news.gallup.com/poll/203198/presidential-approval-ratings-donald-trump.aspx), November 2019 Presidential approval for Trump was 90% amongst Republicans and 4% amongst Democrats. Indicating Trump support versus opposition highly aligned with political party at the time of our field experiment – which is precisely how we then chose to operationalize partisanship in order to manipulate shared partisanship in our study.

That said, it is interesting to consider how other operationalizations of partisanship may impact the effect of shared partisanship on correction engagement. We note this, as well as our above points, in our revised discussion section.

"Second, given the time in which we conducted our field experiment, we operationalized account partisanship primarily through candidate preference. Given that candidate preference at the time we began our field experiment was highly indicative of partisanship [50], we posit that this is an effective way to manipulate shared or misaligned partisan preferences. That said, further research may examine how different representations expressing partisan preference (e.g., different candidate preferences; statements about party or issue position preferences) may differentially impact perceived shared partisanship and subsequent correction engagement." (p. 14)

(5) Regarding the statistical analyses, I think that while Authors tried to be exhaustive in their tests and results in the SI —with almost 100 supplementary tables (too many in my opinion) and 8 supplementary figures— there are some crucial issues in the manuscript itself. There

are small reference errors, such as in p. 5 in which it is stated that the 12 variations of the corrective messages are in SI Table 59, but these are in SI Table 63.

We are grateful to Reviewer 2 for catching this error – it has been corrected accordingly.

"…see SI Table 63 in S1 Supporting Information for all 12 variations of the corrective messages we used)." (p. 4)

(6) And, above all, there are decisions that are not well explained. For instance, I did not understand the binning process for extreme partisanship in Survey Experiment 1. Does it refer to quartiles given that there are four bins? In that case, I'm not sure if the highest quartile could be called extreme partisanship, with the analyses based on this binning seemingly being too arbitrary.

We thank Reviewer 2 for encouraging us to better explain the binning process and analyses for our exploratory extreme partisanship analyses. Participants in our survey experiment are binned such that individuals who reported extreme scale points of partisanship (1 or 7 on our 7-point partisanship measure) are characterized as extreme partisans (as they selected the most extreme partisanship options). About 28% of our participants are extreme partisans in our survey sample as per this metric. As a best proxy for this in our field data, we created bins of users from the absolute value of their inferred partisanship score (0 to 2.5), and classified the highest bin (1.88 to 2.5) as extreme partisans, as this also roughly translated to selecting the highest quarter of extreme partisans using an interval binning similar to our Likert binning in our survey experiment. That is, an inferred score of 1.88 to 2.5 reflects very strong partisanship as per the partisan inference metric from Barberá et al. (2015), and selecting a 1 or 7 scale extreme point for partisanship reflects strong partisanship on our partisan Likert scale.

We do also want to emphasize that these analyses are purely exploratory and are not pre-registered – we conducted them to further try and examine how social connection and shared partisanship may be interrelated beyond our pre-registered analyses.

We have revised the text to clarify these above points.

"However, we also investigated whether treatment effects may have differed for the most politically extreme users in our sample (i.e., 1 or 7 on 7-point partisanship measure; N=222, 27.5% of total sample). In an exploratory, non-pre-registered analysis…" (p. 9)

"To match our survey experiment analyses, we classified users into four bins based on partisan extremity (binned by inferred absolute value of partisanship [25] intervals of 0.625, from 0 to 2.5) and compared the most extreme users (highest bin: partisan score 1.88 to 2.5) to all other users (N=355 high political extremity, 24.4% of total sample) in order to again compare the roughly quarter most politically extreme users to all other users." (pp. 9-10)

"These results provide suggestive evidence that for less politically extreme users, social connection is beneficial overall, whereas for more politically extreme users, social connection

is only beneficial among co-partisans – though we again note that these analyses examining high versus low politically extreme users are purely exploratory." (p. 10)

(7) The rationale to conduct certain analyses and tests is not given, particularly the use of both Bayesian and non-Bayesian regression.

We have revised our manuscript to clarify the reasoning behind our methodological choices. Namely, (a) linear models were pre-registered as our main analyses and are the primary analyses reported throughout the text, given OLS's robustness, flexibility, and high level of interpretability, and (b) exploratory Bayesian regression analyses were conducted and reported as additional robustness checks around the estimation of null hypotheses to add more clarity as to the precision of these null effects.

"…we find via a linear probability model (as pre-registered; linear models used as primary analyses in the current work unless stated otherwise)…" (p. 5)

"…using Bayesian regression analysis [throughout the current work, we perform exploratory Bayesian regression analyses to provide additional robustness checks around our precise estimation of null hypotheses]…" (p. 5)

(8) In addition, the analyses of Likert data are done treating these as interval data, whereas I would argue that it is more appropriate to treat them as ordinal (Göb et al., 2007), albeit this is open to debate. In either case, I think that the graphical representation in Fig. 5 is not suitable for the Likert data at hand; I suggest using instead a diverging stacked bar chart. For the R ecosystem, there are the Likert-related functions of the "HH" package or, if using "ggplot", the package "likert".

• Göb, R., McCollin, C., & Ramalhoto, M. F. (2007). Ordinal methodology in the analysis of Likert scales. Quality & Quantity, 41(5), 601-626.

We thank Reviewer 2 for raising this point about treating Likert data as interval versus ordinal. We contend (and pre-registered) that our scales of obligated responding and perceptions of a norm of reciprocal responding should best be interpreted as interval, with equidistance between our scale points – and thus we report the results of our linear model analyses in the main text. And accordingly, in Figure 5 we plot the mean and 95% confidence intervals of this continuous outcome by social and correction conditions. This graphical representation also helps provide visual synergy in demonstrating the causal effects of social condition here as compared to in our field experiment and survey experiment 1.

However, we appreciate Reviewer 2's suggestion of treating these data as ordinal and plotting these data using a diverging stacked bar chart. As a robustness check, we fit ordinal logistic regression models in addition to our pre-registered linear models for survey experiment 2. Consistent with our primary results, we find that social connection increases reported personal obligation to reply ($b=0.484$, $p<.001$) and nominally increases perceptions of a norm of reciprocal responding ($b=0.214$, $p=.091$) as assessed via ordinal logistic regression models. These results are reported in full in SI Tables 35b and 36b. We also plot these data using

diverging stacked bar charts (from the HH package as suggested) – these visualizations are available in SI Figure S1.

(9) Speaking of R, while I really appreciate that Authors have published the code used for the analyses, in my opinion the way it is structured and not documented greatly hinders reproducibility, and casts doubts on the way the analyses were conducted. For instance, there is no README to guide the interested reader throughout the different files or the libraries needed (and why). Among several things, it is cumbersome to navigate the files and code; it is difficult to discern what is exploratory and what is not; what is the order of the analyses; some notebooks have a few comments explaining a given step but others not at all. For instance, in "SnopeFieldReplies.Rmd" there is a section in which several values are manually assigned, for which "Fix NAs" is the only comment; why are these values missing and how were they recovered? There is also the use of the function "setwd" in several Rmd notebooks that change the working directory to a location present only within the workstation of the original analyst, instead of using a relative path that should work across different workstations.

We appreciate Reviewer 2 encouraging us to better structure and document our reproducibility files. We have revised our OSF files accordingly.

First, as suggested, we have added a README file to better guide interested readers through different folders and files. Additionally, we have renamed files to more intuitively match their relation to the main text and supporting information documents. We believe this README file and our renamed and reorganized OSF documents will greatly improve navigation and understanding of our data and analysis code.

Second, we have gone through our analysis files and more clearly documented discrete analysis steps and procedures, as well as highlight which analyses are pre-registered and which are exploratory. As now noted in the README, the order of analyses in each analysis file is now the order they appear in the codebook.

Third, we have fixed the code in the file "SnopeFieldReplies.Rmd" that required manual updating of computed NA values. The initial code was structured to compute the proportion of raters who evaluated a given field correction reply as demonstrating "resisting information" – but the initial code improperly dealt with NAs for cases of unanimous reporting (hence why NAs were previously manually fixed depending on which way the unanimous rating classified the post as evident from the response coding data – either 100% resisting information or 100% belief updating). We have fixed this bug and updated our code such that no manual NA recoding is required (see lines 45-48 in R markdown file) – all classifications remain identical as initially coded.

Fourth, we have removed the use of absolute file paths requiring "setwd" – we now use the "here" library and here() function to set the file path to the current working directory, and have set up our OSF files such that each analysis file shares the same directory as the data files it requires. This is also specified now in our README file.

(10) On a different note, an important difficulty and aspect of the field experiment is the blocking of some of the bot accounts by targeted counter-partisan users, which as noted by Authors, impairs the "ability to assess whether, and how, they would have engaged with the correction had they received it". I found this phenomenon to be interesting and worth of further investigation to improve the current manuscript. However, I noticed that three of the four Authors have already recently published a research report on this very topic (Martel et al., 2024), which I found interestig and with a more straightforward approach compared to the current manuscript. This other paper, however, brought to my attention that the cited literature in the current manuscript is not the most recent on the topic, with the year 2022 being the latest one (3 out of 33 references).

• Martel, C., Mosleh, M., Yang, Q., Zaman, T., & Rand, D. G. (2024). Blocking of counter-partisan accounts drives political assortment on Twitter. PNAS nexus, 3(5), page 161.

We appreciate Reviewer 2's kind words about our recent research further examining the topic of blocking counter-partisan accounts on Twitter (X). We have edited our manuscript when discussing the issue of blocking in our correction field experiment to inform readers of this subsequent work further investigating partisanship and user blocking.

"The message failure rate was specifically substantially higher in the social counter-partisan condition, likely driven in large part by users blocking the counter-partisan bot after it followed them (subsequent Twitter (X) field research has indeed found that users are 12 times more likely to block counter-partisan accounts compared to co-partisan accounts [33]." (p. 5)

We have also revised our manuscript in several places to cite more recent literature on misinformation correction. These include the following.

-A recent review of the effective correction of misinformation: Prike, T., & Ecker, U. K. (2023). Effective correction of misinformation. *Current Opinion in Psychology*, 101712.

"Numerous survey experiments indicate that corrections generally increase overall belief accuracy [4–6] while almost never backfiring by increasing belief in the debunked claim [7, 8]." (p. 1)

-A recent empirical work showing how corrections are limited in their scope of impact: Carey, J. M., Chun, E., Cook, A., Fogarty, B. J., Jacoby, L., Nyhan, B., ... & Sweeney, L. (2024). The narrow reach of targeted corrections: No impact on broader beliefs about election integrity. *Political Behavior*, 1-14.

"Typical one-off approaches for correcting misinformation have reliable but ultimately small and limited effects in magnitude and scope [17, 18]…" (p. 2)

-Recent works showing that correction sophistication and format have limited impacts on correction efficacy: Badrinathan, S., & Chauchard, S. (2024). "I Don't Think That's True, Bro!" Social Corrections of Misinformation in India. *The International Journal of Press/Politics*, *29*(2), 394-416.; Tulin, M., Hameleers, M., De Vreese, C., Opgenhaffen, M., &

Wouters, F. (2024). Beyond Belief Correction: Effects of the Truth Sandwich on Perceptions of Fact-checkers and Verification Intentions. ***Journalism Practice*****, 1-20.**

"For the correction reply itself, although research has found no significant effect of the tone of corrective messages on intentions to reply versus ignore [12–14] nor substantial differences in the sophistication or formatting of corrections [31, 32]…" (p. 4)

-A recent paper on the importance of source credibility and source discreditation on corrections: Ecker, U. K., Prike, T., Paver, A. B., Scott, R. J., & Swire-Thompson, B. (2024). Don't believe them! Reducing misinformation influence through source discreditation. ***Cognitive Research: Principles and Implications*****,** ***9*****(1), 52.**

"This would be consistent with recent work on the importance of source credibility on correction efficacy – signaled counter-partisanship may incidentally discredit the correction source for extreme partisans [38]." (p. 12)

-A recent paper on using LLMs to develop a model predicting how users will respond to social corrections: He, B., Ma, Y., Ahamad, M., & Kumar, S. (2024, May). Corrective or Backfire: Characterizing and Predicting User Response to Social Correction. In ***Proceedings of the 16th ACM Web Science Conference*** **(pp. 149-158).**

"Furthermore, recent advancements in large-language models have allowed for the development of user response prediction models designed to identify whether a social correction message will be beneficial neutral, or backfire [39]. A critical component of these models is training data consisting of existing responses to social corrections – and thus eliciting greater feedback and engagement with online corrections is integral for further developing such models and learning more about the features of corrections that predict corrective versus neutral or backfire effects. Our current work suggests that minimal contextual features such as establishing a minimal social connection via following soon-to-be corrected users can increase engagement with social corrections – thus allowing these models and researchers to learn more about how these corrections were received by users." (pp. 12-13)
